# Experience-dependent emergence of multi-scale spatial organization during foraging in freely moving marmosets

**DOI:** 10.1016/j.isci.2026.117294

**Published:** 2026-08-26

**Authors:** Nada El Mahmoudi, Francesca Lanzarini, Farzad Ziaie Nezhad, Deepak Surendran, Jean Laurens

**Affiliations:** 1 Ernst Strüngmann Institute (ESI) of the Max Planck Society, 60528 Frankfurt am Main, Germany

**Keywords:** spatial navigation, depletion-based foraging, common marmoset, semi-naturalistic behavior, multi-scale spatial organization, successor representation, spatial cognition

## Abstract

How spatial organization emerges through experience in freely moving primates remains incompletely understood. We addressed this question in two common marmosets foraging in a semi-naturalistic depletion-based environment with a fixed spatial layout. With experience, animals increasingly directed choices toward yet-unvisited locations, largely accounted for by the consolidation of a stable transition scaffold, alongside sensitivity to the current depletion state within each foraging episode. This scaffold organized navigation at multiple scales: local transitions became increasingly reliable; complete visitation sequences remained variable, reflecting flexible recombination of local transitions; and the current location predicted visits over multiple subsequent steps, revealing a state-dependent multi-step predictive organization that became increasingly consistent with experience. Together, these findings provide behavioral evidence that multi-scale spatial organization can emerge with repeated foraging experience in freely moving marmosets, and establish a longitudinal, semi-naturalistic behavioral and analytical framework for relating ecological foraging behavior to theoretical and computational accounts of spatial cognition.

## Highlights

• Marmosets increasingly direct choices toward yet-unvisited food locations
• Visitation choices are sensitive to the current depletion state
• Stable local transitions coexist with flexible visitation sequences
• Current location predicts which food locations are visited over several steps

## Graphical abstract

**Figure:**
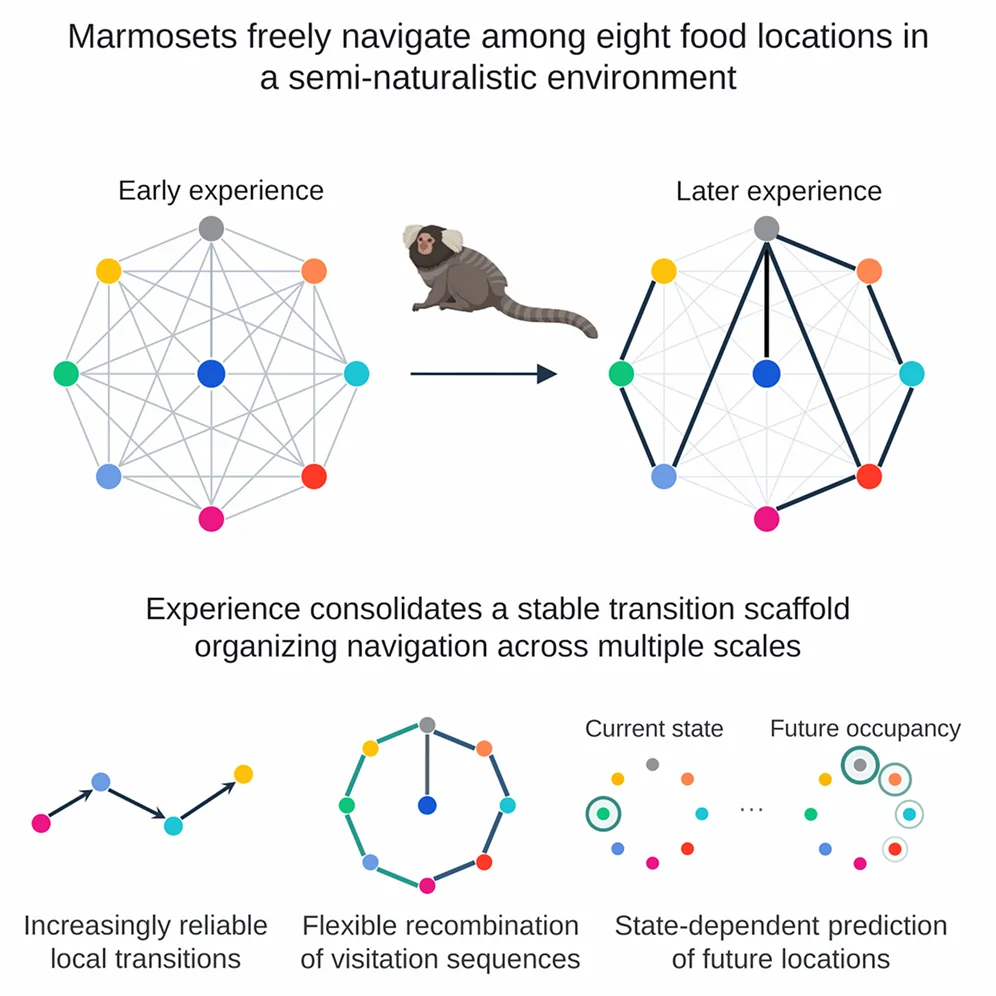

## Teaser

Behavioral neuroscience; Cognitive neuroscience

## Introduction

Understanding how organized behavior emerges from continuous interaction with the environment is a central challenge in naturalistic neuroscience and behavioral ecology.^1^^,^^2^ Spatial navigation is a powerful model for this question, because behavioral organization is directly expressed in movement trajectories and can be tracked as it changes.

Spatial navigation can be organized in multiple ways, depending on how locations and their relations are represented and used to guide behavior. This organization is not fixed: it depends on environmental structure, task demands, and the information available, implying that experimental paradigms actively shape the organization that is observed.^3^ In particular, navigation may rely on route-based, topological, or metric forms of spatial organization, which differ in the nature of the spatial relations they capture.^3^^,^^4^^,^^5^^,^^6^^,^^7^ This raises a key question: how does spatial organization develop when external constraints are weak, and behavior is primarily self-guided?

Foraging provides an ethologically grounded way to address this question. During foraging, animals repeatedly sample familiar environments while searching for spatially distributed resources, naturally coupling navigation, memory, and learning. Accordingly, foraging has been proposed as a unifying framework linking simplified laboratory tasks and unconstrained natural behavior.^8^

This ethological framing is especially relevant for non-human primates (NHPs), whose food acquisition often depends on navigation in spatially complex environments. In the wild, NHPs show flexible foraging routines supported by spatial memory.^9^ Yet capturing how such organization emerges over time remains challenging: laboratory paradigms often constrain movement and reduce navigation to screen-based decisions, whereas field studies preserve natural behavior but typically lack the temporal resolution and experimental control needed to track how organization develops across repeated experience. As a result, how spatial organization develops in freely moving NHPs through repeated experience remains poorly understood.

The common marmoset *(Callithrix jacchus*) provides a particularly well-suited model to bridge this gap. As small arboreal foragers, marmosets naturally navigate complex three-dimensional environments and rely on spatial memory to exploit spatially distributed food resources.^10^^,^^11^^,^^12^^,^^13^^,^^14^ Their ethology, combined with their suitability for freely moving laboratory paradigms, makes them a valuable model for investigating how spatial organization emerges through foraging experience.

Classic approaches to studying foraging include depletion-based paradigms, in which revisiting an already exploited resource yields no benefit and incurs energetic costs, consistent with core principles of optimal foraging theory.^15^^,^^16^^,^^17^^,^^18^ Because the depletion contingency captures a challenge animals routinely face in nature, namely spatially distributed resources that cannot be profitably revisited once exploited, such designs allow spatial organization to emerge from the foraging problem itself rather than from explicit training demands.

Here, we adapted depletion-based foraging to a semi-naturalistic environment in which two common marmosets freely navigated among a fixed configuration of eight spatially distributed food dispensers. Combining behavioral clustering, transition analysis, and probabilistic modeling across thirty sessions, we tracked how spatial behavior became progressively organized through repeated foraging experience. Animals increasingly directed choices toward yet-unvisited locations, a change that reflected two complementary components: a dominant transition scaffold between food locations that consolidated across sessions, and a second component reflecting sensitivity to which locations had already been depleted within each foraging episode. This scaffold supported a multi-scale organization of navigation in which local transitions became increasingly reliable, whereas complete foraging sequences remained variable. At the same time, the current location predicted the distribution of visits over multiple subsequent steps, revealing a multi-step predictive organization that became increasingly stable with experience.

Collectively, these results provide behavioral evidence that multi-scale spatial organization can emerge with repeated foraging experience in freely moving marmosets, and offer a longitudinal, semi-naturalistic behavioral and analytical framework for studying how ecological foraging behavior relates to theoretical and computational accounts of spatial cognition.

## Results

**Figure 1:**
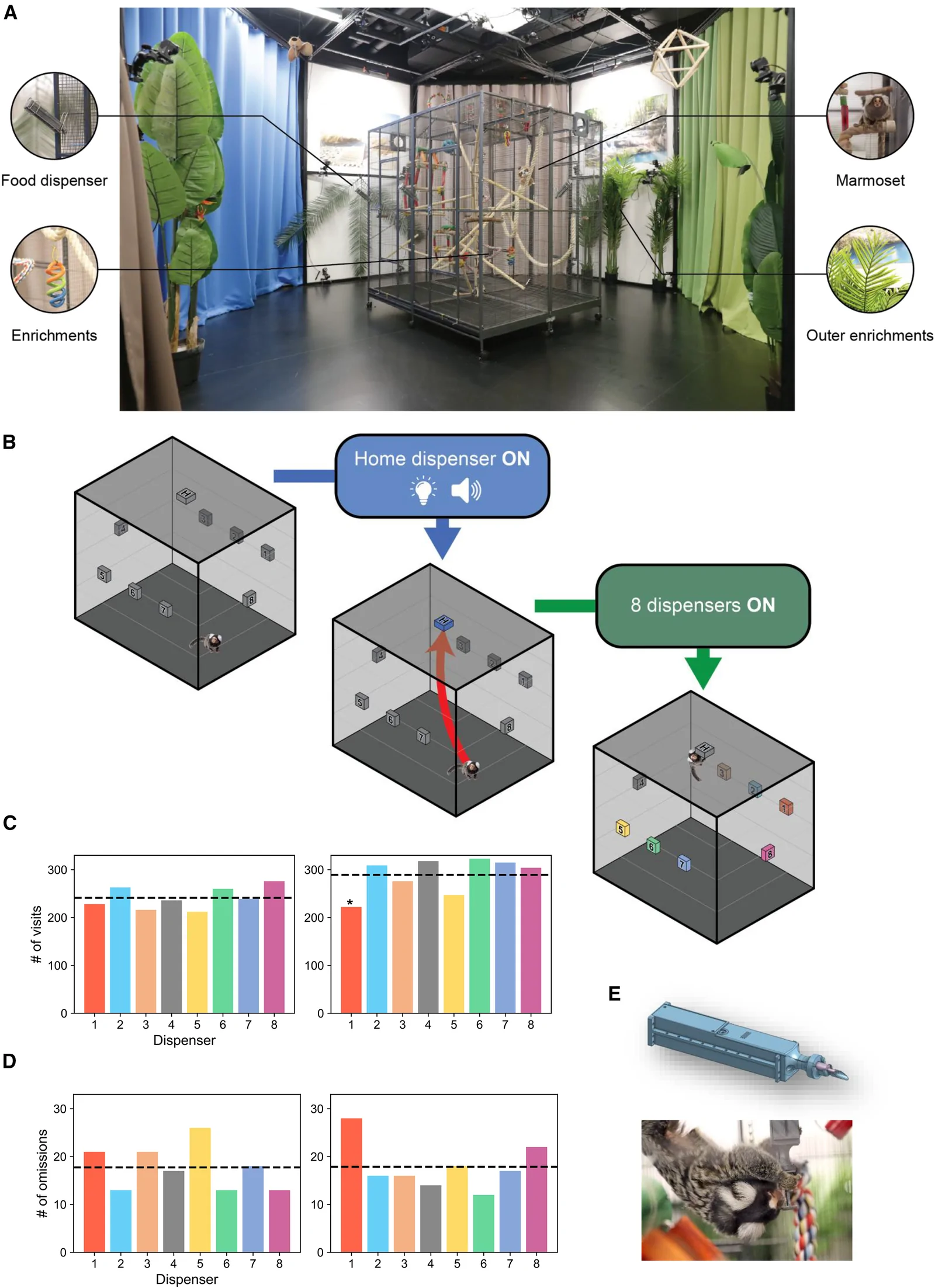
A semi-naturalistic foraging environment for freely moving marmosets (A) Experimental setup. The experimental environment consisted of a wire-mesh enclosure (1.8 m × 1.4 m × 1.6 m; ∼4 m^3^) that allowed marmosets to move freely in three dimensions. The enclosure contained multiple structural enrichments, including wooden sticks, semi-rigid ropes, platforms, and suspended objects, supporting arboreal locomotion and exploration. The environment also included external enrichments, such as colored curtains, landscape posters, and large artificial plants, which remained fixed across sessions. An overhead action camera recorded behavior throughout the experiment. A food dispenser designated as the home dispenser (HD) was mounted centrally on the ceiling, and eight additional food dispensers were evenly distributed around the perimeter of the enclosure. (B) Trial design. At the beginning of each trial, the HD was activated and signaled by auditory and visual cues. Once the animal collected the reward at the HD, all eight peripheral dispensers became active simultaneously. Each peripheral dispenser delivered a single aliquot of gum Arabic per trial, such that only the first visit to a given location was rewarded, introducing a depletion contingency. A trial ended once all eight peripheral dispensers had been visited at least once, or after a maximum duration of 10 min. (C) Total visits per dispenser. Bar plots show the total number of visits to each of the eight peripheral dispensers for Animal E (*left*) and Animal F (*right*), pooled across sessions. The dashed horizontal line indicates the expected number of visits under a uniform distribution. Deviations from uniformity were assessed using a chi-squared test, followed by binomial post hoc tests comparing each dispenser to the expected proportion (Bonferroni-corrected; ∗*p* < 0.05). (D) Total omissions per dispenser. Bar plots show the total number of omissions (dispensers not visited within a trial) for each of the eight peripheral dispensers for Animal E (*left*) and Animal F (*right*), pooled across sessions. The dashed horizontal line indicates the expected number of omissions under a uniform distribution. Deviations from uniformity were assessed using a Chi-squared test. (E) Reward dispenser design. Design of the custom-built food dispenser used in the experiment (*top*), and an example of a marmoset consuming gum Arabic from the HD (*bottom*). See also Figure S1 and Table S1.

We studied spatial behavior in two common marmosets using a depletion-based foraging paradigm implemented in an enriched, semi-naturalistic environment (Figure 1A). Animals moved freely within a large arena (1.8 m × 1.4 m × 1.6 m; ∼4 m^3^) and accessed food rewards (gum arabic, a palatable liquid reward not included in the animals’ standard daily diet) from eight peripheral dispensers arranged around its perimeter (Figure 1E).

The paradigm was organized into trials, each constituting a depletion episode. Trials were initiated by activation of the central “home” dispenser, signaled by auditory and visual cues, and accompanied by reward delivery (Figure 1B). Upon visiting this dispenser, all eight peripheral dispensers delivered a single aliquot of gum arabic. Animals consumed the full aliquot upon first contact, as verified during habituation. A trial ended once all eight peripheral locations had been visited at least once, regardless of any revisits along the way, or after a maximum duration of 10 min. Upon completion of a trial, a new trial was initiated by activating the home dispenser.

Sessions were conducted on separate days, typically 24 h apart, with longer intervals over weekends. All sessions took place between the animals’ two daily meals, a period during which marmosets are naturally motivated to forage. Each subject completed 30 sessions, yielding 233 trials for Animal E and 232 trials for Animal F.

### Marmosets broadly sample space while expressing heterogeneous responses to depletion

In our paradigm, marmosets were free to decide whether to forage, which locations to visit, and in what order. We therefore began by quantifying how animals interacted with the spatial layout and the depletion contingency.

#### Marmosets interact robustly with food dispensers in a semi-naturalistic foraging environment

We first asked whether marmosets sampled the full spatial layout of the arena. To this end, we examined the distribution of visits and omissions (i.e., locations not visited within a trial) across the eight peripheral dispensers, pooling data across sessions to assess overall spatial coverage (Figures 1C and 1D). Both animals showed significant deviations from uniformity in visit distributions (chi-squared test; Animal E: *χ*^2^ = 15.47, *p* = 0.03; Animal F: *χ*^2^ = 33.6, *p* < 0.001), although post-hoc binomial tests indicated that only Animal F visited one dispenser less often than expected (Bonferroni-corrected *p* < 0.05). In contrast, omissions did not deviate from uniformity in either animal (Animal E: *χ*^2^ = 8.9, *p* = 0.26; Animal F: *χ*^2^ = 9.89, *p* = 0.19), indicating no systematic neglect of specific locations.

Overall, both animals sampled all dispensers broadly, providing a basis for subsequent analyses of depletion-related spatial behavior.

#### Non-rewarded events decrease with experience but reflect dissociable interaction patterns

To characterize how animals interacted with the depletion contingency, we quantified three types of non-rewarded events. These comprised revisits to already-sampled peripheral dispensers, omissions (peripheral dispensers not visited within a trial), and returns to the home dispenser before all peripheral locations had been sampled. Across sessions, the mean number of non-rewarded events decreased significantly in both animals (Figures S1A and S1B; Table S1), indicating a progressive adjustment of behavior to the depletion structure.

Importantly, this global decrease masked marked differences between event types. Revisits, omissions, and home returns followed distinct temporal trajectories over experience and exhibited different covariation patterns at both the session and trial levels (Figures S1C–S1F).

Together, these results show that although non-rewarded events decreased over experience, revisits, omissions, and home returns reflected distinct interaction patterns with the depletion contingency rather than a single homogeneous category.

### Trial-by-trial variability reflects discrete execution profiles

Because non-rewarded events directly reflected how animals interacted with the depletion contingency and showed substantial heterogeneity at both session and trial levels, we next asked whether trial-by-trial behavior could be grouped into a small number of consistent execution profiles.

#### Unsupervised clustering identifies reproducible trial execution profiles

We applied an unsupervised k-means clustering analysis to identify patterns of trial execution. Clustering was performed on the total number of dispenser visits and the number of omissions, capturing complementary dimensions of how animals interacted with the depletion contingency.

**Figure 2:**
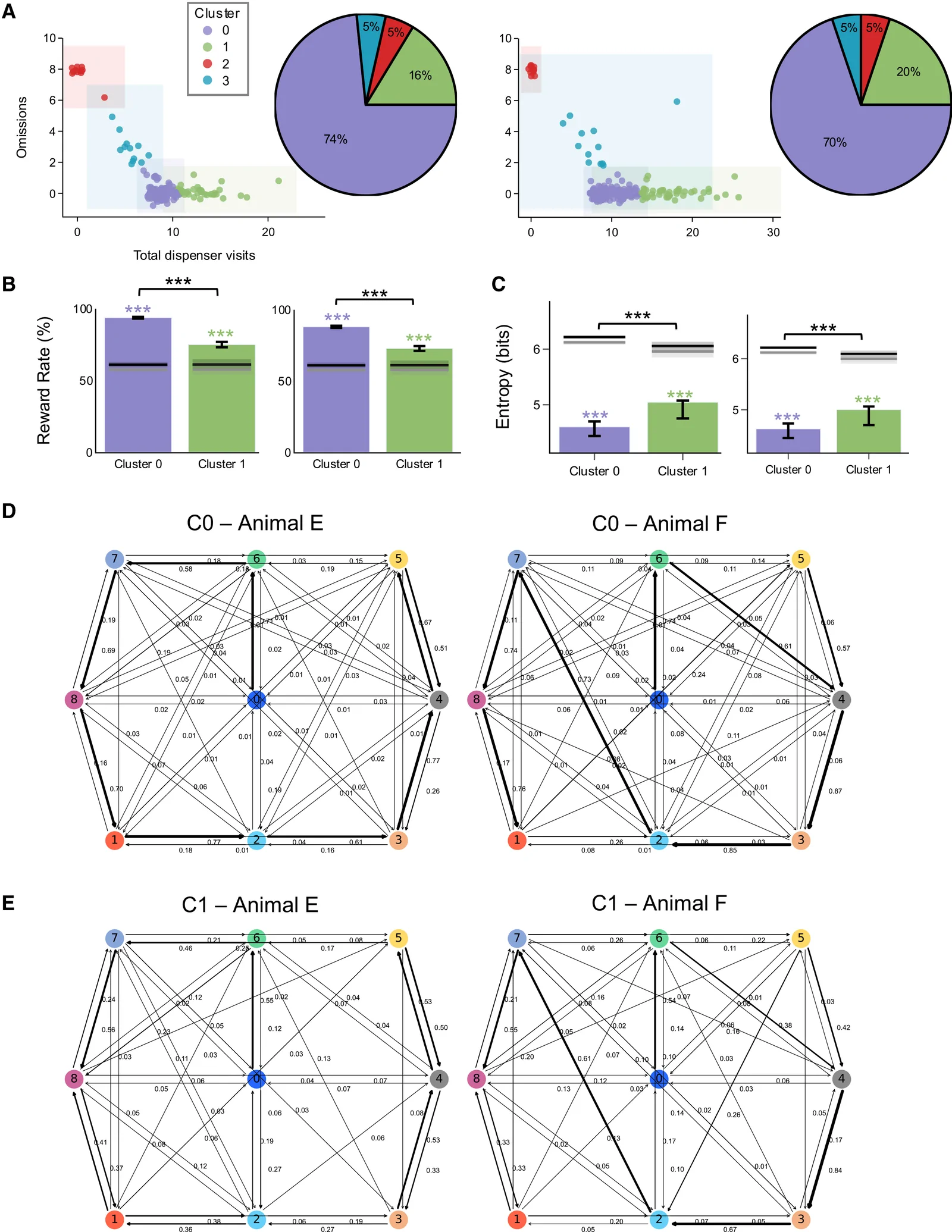
Trial-by-trial clustering reveals discrete execution profiles (A) Unsupervised clustering of trial execution. Scatter plots showing the results of the K-means clustering analysis for Animal E (*left*) and Animal F (*right*). Clustering was performed separately for each animal. Each point represents a single trial, plotted as a function of the total number of dispenser visits and the number of omissions, and colored according to cluster identity (Clusters 0-3). Data points are jittered for visibility. Shaded regions indicate the approximate extent of each cluster in feature space. Pie charts summarize the proportion of trials assigned to each cluster for each animal. (B) Reward rate across dominant execution profiles. Bar plots showing the mean reward rate during the first eight choices of each trial for Clusters 0 and 1 in Animal E (*left)* and Animal F (*right*). Error bars indicate the standard error of the mean (SEM). Solid reference lines indicate null expectations derived from the uniform random-choice (black) and spatially constrained random-walk (gray) models, with null distributions resampled with replacement to match the number of empirical trials in each cluster and animal. Shaded regions indicate the corresponding 95% confidence intervals for null distributions. Statistical comparisons between clusters were performed using two-sided Mann-Whitney U tests. Comparisons between each cluster and its corresponding reference models were performed using Monte Carlo tests with Bonferroni correction. Colored asterisks indicate comparisons to reference models, and black asterisks indicate between-cluster comparisons (∗∗∗ p < 0.001). (C) Global transition entropy across dominant execution profiles. Bar plots showing the mean global entropy of dispenser-to-dispenser transitions for Clusters 0 and 1 in Animal E (*left*) and Animal F (*right)*. Error bars represent bootstrap-estimated 95% confidence intervals obtained by resampling trials within each cluster. Solid reference lines and corresponding shaded regions indicate null expectations and 95% confidence intervals derived from the stochastic reference models as in panel B. Statistical comparisons follow the same conventions as in panel B. (D) Transition maps for Cluster 0. Transition maps visualizing the dispenser-to-dispenser transition matrices for Cluster 0 (C0), computed from the first eight choices of each trial, corresponding to the maximum number of potentially rewarded peripheral visits, for Animal E (*left*) and Animal F (*right*). Nodes correspond to dispenser locations arranged according to their spatial positions in the environment. Directed edges represent transition probabilities, with line thickness proportional to transition weight. (E) Transition maps visualizing the corresponding dispenser-to-dispenser transition matrices for Cluster 1 (C1) for Animal E (*left*) and Animal F (*right*), using the same conventions as in panel D. See also Figure S2 and Table S2.

For both animals, a four-cluster solution (Figure 2A) yielded well-separated and coherent groups, as assessed by silhouette score and inertia (Table S2). The resulting clusters corresponded to distinct trial execution profiles (Figure S2A).

Cluster 0 comprised trials with approximately eight unique dispenser visits, near-zero non-rewarded events, and short durations, such that all peripheral dispensers were sampled within the depletion episode with few additional visits. Cluster 1 also involved sampling of all eight dispensers, but was associated with frequent revisits and home returns, leading to longer trial durations before completion. In contrast, Cluster 2 was dominated by high omission rates and limited peripheral sampling, whereas Cluster 3 exhibited intermediate levels of visits and omissions; in both cases, one or more dispensers remained unvisited at trial termination.

Clusters 0 and 1 together accounted for 90% of trials in both animals, whereas Clusters 2 and 3 were less frequent (10%). We next examined how the relative frequency of trials in each cluster evolved across sessions (Figures S2B and S2C). The proportion of Cluster 0 trials increased with session number, whereas Cluster 1 decreased; by contrast, Clusters 2 and 3 remained stable over time.

Together, these analyses show that trial-by-trial behavior can be grouped into a small number of reproducible execution profiles, with the two dominant profiles (Clusters 0 and 1) exhibiting complete sampling and experience-dependent shifts in prevalence.

#### Dominant trial profiles differ quantitatively within a shared transition structure

Because Clusters 0 and 1 were the only execution profiles that both involved complete sampling of all dispenser locations and changed with experience, subsequent analyses focused on these two profiles. We asked whether the differences in trial execution identified above were associated with differences in dispenser-to-dispenser transition structure, i.e., the pattern of successive moves between dispensers across trials. Specifically, we asked whether these profiles reflected qualitatively distinct sets of transitions, or instead quantitative differences in how a common set of transitions was weighted.

To establish a common analysis window across trials, we focused on the first eight choices of each trial, corresponding to the maximum number of potentially rewarded peripheral visits. We compared Clusters 0 and 1 directly and evaluated early dispenser choice patterns relative to two stochastic reference models: a uniform random-choice model selecting among all dispensers with equal probability, and a spatially constrained random-walk model in which choices were random but weighted by spatial proximity (see STAR Methods).

We quantified reward rate, defined here as the proportion of early choices directed to dispensers still containing food (i.e., not yet sampled within the ongoing trial). For both animals, reward rate was significantly higher in Cluster 0 than in Cluster 1 (Figure 2B; two-sided Mann-Whitney U test, *p* < 0.001). In addition, reward rates in both clusters exceeded those expected under the stochastic reference models (Monte Carlo tests; Bonferroni-corrected, *p* < 0.01).

We next examined whether the global structure of dispenser-to-dispenser transitions differed across clusters. Global transition entropy, which quantifies how broadly transition probabilities are distributed across all dispenser pairs, was significantly higher in Cluster 1 than in Cluster 0 (Figure 2C; two-sided permutation tests; Animal E, *p* = 0.04; Animal F, *p* = 0.01), although entropy remained lower than stochastic expectations in both clusters (Monte Carlo tests; Bonferroni-corrected, all *p* < 0.01).

Despite this difference, dispenser-to-dispenser transition matrices were highly similar between Clusters 0 and 1 for both animals (Figures 2D and 2E; Figure S2D), indicating reliance on largely overlapping sets of transitions. Differences between clusters primarily reflected quantitative changes in transition weighting rather than changes in transition identity.

Finally, we examined transition-level contributions to revisits and home returns (Figures S2E and S2F). Nearly all non-rewarded events in Cluster 1 arose from transitions also present in Cluster 0, and transition-specific non-rewarded probabilities were strongly correlated across clusters. However, these shared transitions were weighted differently: Cluster 1 placed greater weight on transitions associated with higher non-rewarded probability, resulting in a higher overall likelihood of encountering non-rewarded outcomes.

Taken together, these results indicate that Clusters 0 and 1 did not reflect qualitatively distinct navigational structures, but rather quantitative differences in how a shared transition repertoire was weighted.

### Experience-dependent refinement of the transition repertoire

Having shown that Clusters 0 and 1 were the only dominant execution profiles that both completed the depletion episode and changed with experience, and that they relied on a shared transition repertoire differing primarily in weighting rather than identity, we next asked how this common transition structure emerged and changed with experience. To address this question, we pooled trials from Clusters 0 and 1 and analyzed behavior across six consecutive five-session blocks spanning early, intermediate, and late experience (Beg 1: sessions 1–5; Beg 2: 6–10; Int 1: 11–15; Int 2: 16–20; Last 1: 21–25; Last 2: 26–30).

#### Transitions become progressively more selective across session blocks

**Figure 3:**
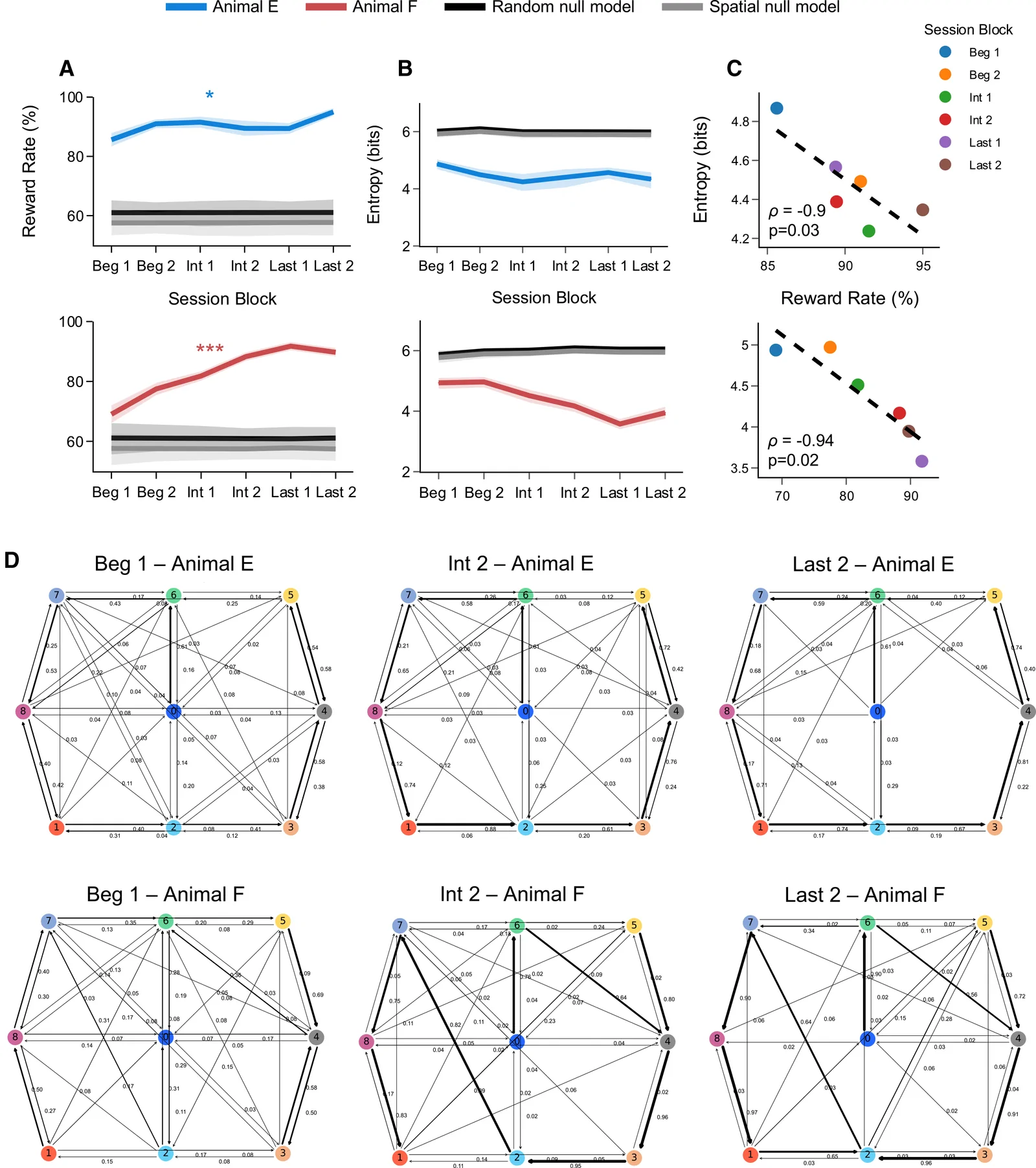
Experience-dependent refinement of the transition repertoire (A) Reward rate increases across session blocks. Line plots showing the mean reward rate during the first eight choices of each trial across six consecutive five-session blocks (Beg 1: sessions 1-5; Beg 2: 6-10; Int 1: 11-15; Int 2: 16-20; Last 1: 21-25; Last 2: 26-30) for Animal E (*top*) and Animal F (*bottom*). Colored lines indicate mean reward rate within each session block, and corresponding shaded areas represent the standard error of the mean (SEM) across trials. Reference lines indicate null expectations derived from the uniform random-choice (black) and spatially constrained random-walk (gray) models, with null distributions resampled with replacement to match the number of empirical trials in each block and animal. Corresponding shaded regions indicate the 95% confidence intervals for null expectations. Comparisons between empirical reward rates and reference models were performed using Monte Carlo tests with Bonferroni correction across models. Changes across session blocks were assessed using Kruskal-Wallis tests followed by Dunn post hoc multiple comparisons with Bonferroni correction. Asterisks indicate significant differences across session blocks (∗p < 0.05; ∗∗∗p < 0.001). (B) Global transition entropy across session blocks. Line plots showing the evolution of the global entropy of dispenser-to-dispenser transitions across session blocks for Animal E (top) and Animal F (bottom). Colored lines indicate mean entropy values, and shaded areas represent bootstrap-estimated 95% confidence intervals obtained by resampling trials within each session block. Solid reference lines and corresponding shaded regions indicate null expectations and 95% confidence intervals derived from the stochastic reference models as in panel A. Pairwise differences between session blocks were assessed using permutation tests with Bonferroni correction. (C) Relationship between reward rate and global transition entropy. Scatter plots showing the relationship between block-wise reward rate and block-wise global transition entropy for Animal E (*top*) and Animal F (*bottom*). Each point corresponds to a session block and is color-coded by block identity. Dashed lines indicate linear trends shown for visualization. Spearman’s rank correlation coefficient (ρ) is reported for each animal; statistical significance was assessed using permutation tests, and permutation-based p-values are reported. (D) Transition maps across session blocks. Transition maps visualizing the dispenser-to-dispenser transition matrices computed from the first eight choices of each trial, corresponding to the maximum number of potentially rewarded peripheral visits, for representative session blocks. The top row shows results for Animal E, and the bottom row shows results for Animal F. Nodes correspond to dispenser locations arranged according to their spatial positions in the environment. Directed edges represent transition probabilities, with line thickness proportional to transition weight. See also Figure S3.

We first examined whether repeated experience was associated with changes in early choice behavior under the depletion contingency. As an operational measure of how dispenser choices aligned with the depletion structure, we quantified the reward rate during the first eight choices of each trial. Across all session blocks, empirical reward rates exceeded null expectations derived from stochastic reference models (uniform random-choice and spatially constrained random-walk; Monte Carlo tests, Bonferroni-corrected, all *p* < 0.001; Figure 3A). Beyond this baseline, reward rate increased significantly across session blocks in both animals (Kruskal-Wallis tests; Animal E: *H* = 11.66, *p* = 0.03; Animal F: *H* = 64.6, *p* < 0.001), with late blocks exhibiting higher reward rates than early blocks (post hoc Dunn tests, Bonferroni-corrected, *p* < 0.01), indicating that early choices were increasingly directed toward yet-unvisited dispensers with experience.

To characterize how dispenser-to-dispenser transition probabilities were distributed across session blocks, we quantified the global entropy of transitions within each block (Figure 3B; Figure S3A). Global transition entropy remained consistently lower than stochastic expectations across all blocks (Monte Carlo tests, Bonferroni-corrected, all *p* < 0.001), indicating more concentrated transition probabilities than expected from random or spatially constrained choice alone. Importantly, entropy decreased significantly with experience, with early blocks exhibiting higher entropy than later blocks (permutation tests across session blocks, Bonferroni-corrected, Beg 1 vs. Last 2 *p* < 0.05), consistent with a progressive concentration of transition probabilities.

Across session blocks, reward rate and global transition entropy were negatively correlated (Figure 3C; Spearman’s rank correlation with permutation-based *p* values; Animal E: *ρ* = −0.9, *p* = 0.03; Animal F: *ρ* = −0.94, *p* = 0.02), indicating that blocks with more selective transition structure were also those in which early choices were more consistently aligned with the depletion contingency.

To further characterize how the global reduction in transition entropy was distributed across the spatial layout, we examined local transition entropy for each dispenser. Local entropy analyses revealed location-specific differences in transition selectivity, with additional heterogeneity in experience-dependent refinement in Animal F but not Animal E (Figures S3D and S3E).

#### Transition structure is conserved while transition probabilities sharpen with experience

While the results above indicate a progressive increase in transition selectivity, changes in global entropy alone do not specify how this refinement arises. In principle, a decrease in entropy could reflect either a reorganization of which transitions are expressed or a progressive sharpening of an already established transition repertoire.

Transition maps provided an initial qualitative view of these effects (Figure 3D), revealing the persistence of a common set of dominant transitions across session blocks alongside progressive changes in their relative strengths.

To quantify this observation, we directly compared dispenser-to-dispenser transition matrices across session blocks. Transition matrices were highly correlated across session blocks for both animals (Figure S3B), indicating that the relative structure of dispenser-to-dispenser transitions was already present early and remained stable over experience. In contrast, differences in absolute transition probabilities between blocks progressively decreased across session blocks (Figure S3C), consistent with a stabilization and sharpening of transition weights rather than changes in transition identity.

Together, these results show that experience-dependent changes in navigation reflected a progressive sharpening of transition probabilities within a conserved transition repertoire, yielding increasingly structured behavior under the depletion contingency.

### Within-trial visit history contributes to choice outcomes beyond transition structure

The analyses above showed that experience progressively refines the structure of dispenser-to-dispenser transitions and that this refinement is tightly associated with increases in reward rate. However, reward availability in this paradigm also changes dynamically within each trial as locations are progressively sampled. Consequently, behavior may depend not only on transition structure but also on information available within the ongoing trial about which dispensers have already been visited (i.e., within-trial visit history). We therefore asked whether such information influenced dispenser selection, and to what extent behavior could be explained by transition structure alone.

#### Reward probability varies with trial progression and exceeds structure-only predictions

To characterize how choice outcomes evolved within trials, we examined how the probability of selecting a rewarded dispenser varied as a function of trial progression. For each choice within a trial, we defined *k* as the number of distinct peripheral dispensers already visited at that point and computed *P(reward |k)* within the standardized eight-choice window. We then compared empirical curves to those generated by a structure-only agent, implemented as a first-order Markov model. For each animal, this agent sampled dispensers according to the empirically observed dispenser-to-dispenser transition probabilities but did not track which dispensers had already been visited during the current trial.

To quantify these effects, we fit logistic regression models predicting reward probability as a function of trial progression (*k*), session block, and agent identity (empirical versus structure-only; see Table S3 for full model results).

**Figure 4:**
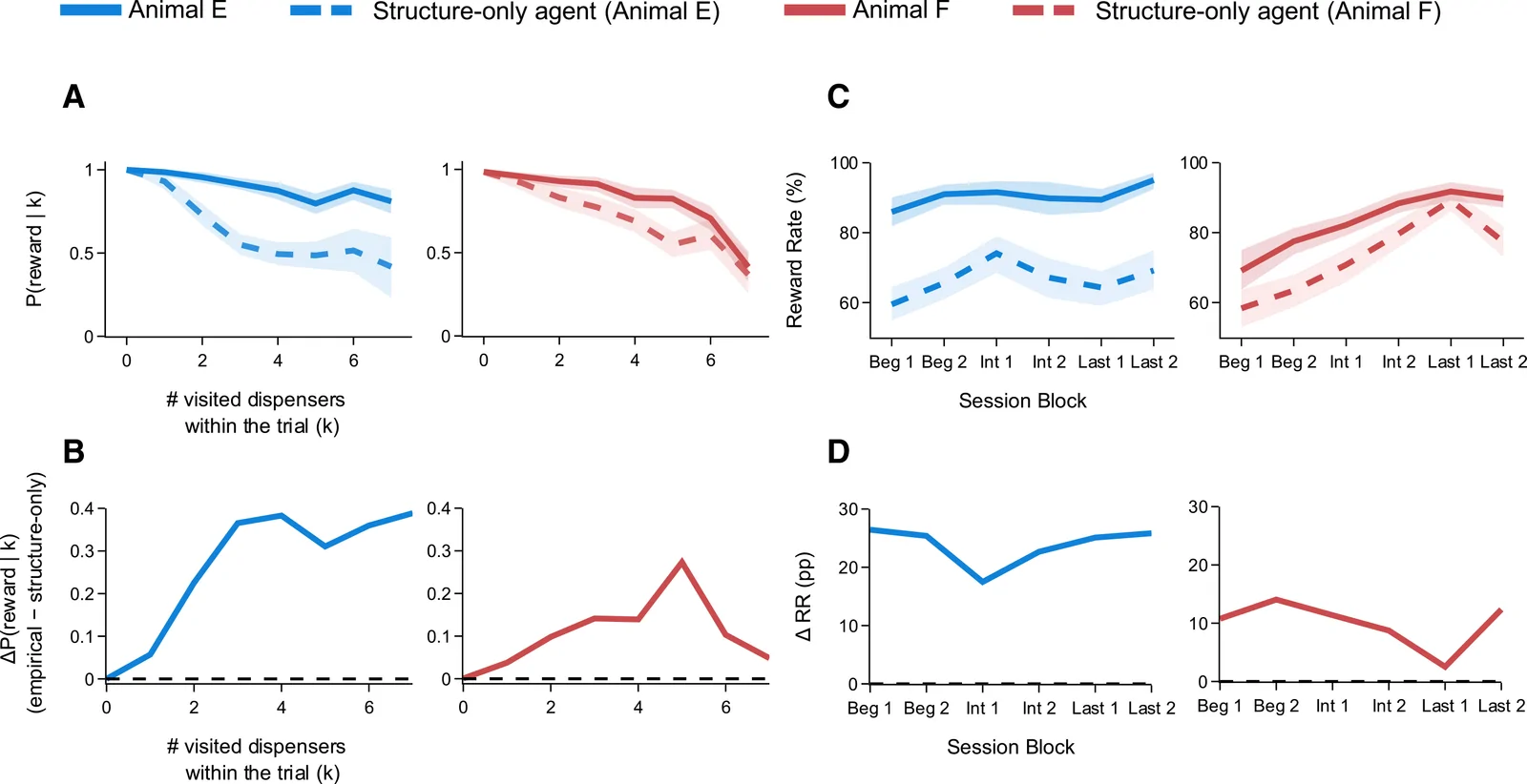
Within-trial visit history contributes to reward probability and reward rate beyond transition structure (A) Reward probability during trial progression. Probability of selecting a rewarded dispenser, *P*(reward | *k*), as a function of trial progression, quantified by the number of distinct peripheral dispensers already visited within the ongoing trial (*k*). Solid lines show empirical curves; dashed lines show predictions from the structure-only agent, implemented as a first-order Markov model constrained by empirically observed dispenser-to-dispenser transition probabilities but lacking access to within-trial visit history, for Animal E (left) and Animal F (right). Shaded regions indicate 95% confidence intervals derived from trial-level bootstrap resampling for empirical curves and matched-*N* resampling for structure-only predictions. (B) Empirical advantage over the structure-only agent during trial progression. Difference between empirical and structure-only reward probability curves, Δ*P*(reward | *k*) = *P*empirical(reward | *k*) − *P*structure(reward | *k*), for Animal E (left) and Animal F (right). Positive values indicate higher empirical reward probability than predicted from transition structure alone. (C) Reward rate across session blocks. Reward rate across session blocks for empirical behavior (solid lines) and the structure-only agent (dashed lines), for Animal E (left) and Animal F (right). Reward rate was defined as the percentage of choices directed to previously unvisited peripheral dispensers within the standardized eight-choice window. Shaded regions indicate 95% confidence intervals derived from trial-level bootstrap resampling for empirical values and matched-*N* resampling for structure-only estimates. (D) Empirical reward-rate advantage across session blocks. Difference in reward rate between empirical behavior and the structure-only agent across session blocks, Δ*RR* = *RR*empirical − *RR*structure, expressed in percentage points, for Animal E (left) and Animal F (right). Positive values indicate an empirical advantage beyond transition structure alone. See also Figure S4 and Table S3.

For both animals, reward probability declined significantly with increasing *k*, indicating robust dependence of choice outcomes on trial progression (Figure 4A; logistic regression, *p* < 0.001). At the same time, empirical reward probabilities remained consistently higher than those generated by the structure-only agent across most values of *k* (Figure 4B). Logistic regression confirmed this difference, showing that reward probability was significantly higher in empirical behavior than in the structure-only agent in both animals (*p* < 0.001). These results indicate that transition structure alone could not fully account for observed choice outcomes during trial progression.

We next asked whether these effects changed with experience. To this end, empirical curves and the corresponding structure-only predictions were recomputed separately within each session block. Logistic regression showed a significant effect of session block in both animals (Animal E: *p* = 0.01; Animal F: *p* < 0.001), indicating higher reward probability in later session blocks. In addition, the interaction between session block and agent identity was not significant in Animal E but was significant in Animal F, indicating that the difference between empirical behavior and the structure-only agent remained stable in Animal E but changed across session blocks in Animal F (Table S3; Figure S4).

Together, these results showed that choice outcomes varied systematically with trial progression and that this dependence was not fully captured by transition structure alone, consistent with an additional contribution from within-trial visit history.

#### A structure-only agent captures a large fraction of empirical reward rate

Although the analyses above indicate an additional contribution beyond transition structure during trial progression, they do not establish how much this contribution affects behavior across complete trials. We therefore asked to what extent the empirical reward rate could be accounted for by transition structure alone. To address this question, we compared empirical reward rates to those generated by the same structure-only agent, evaluated separately for each animal and session block.

Across session blocks, reward rates generated by the structure-only agent remained below empirical values but accounted for a large fraction of the observed reward rate in both animals (Figure 4C). On average, the agent accounted for approximately 74% of the empirical reward rate in Animal E (range: 70–81%) and 88% in Animal F (range: 82–97%), indicating that a substantial fraction of the observed reward rate is compatible with transition structure alone.

Nevertheless, empirical reward rates consistently exceeded those generated by the structure-only agent across all session blocks (Figure 4D). The resulting positive difference (ΔRR = RR_empirical – RR_agent) indicated an additional contribution beyond transition structure alone.

Together, these results suggest that behavior reflected two complementary components: a dominant contribution associated with dispenser-to-dispenser transition structure, and an additional contribution consistent with within-trial visit history.

### Spatial organization combines strong local predictability with sequence-level variability

Because the analyses above established that behavior relies strongly on a structured repertoire of dispenser-to-dispenser transitions, we next asked how this transition structure organizes navigation across different scales of behavior.

To address this question, we first characterized local organization by quantifying how reliably the next dispenser could be predicted from the current location. We then asked whether this local organization was sufficient to account for complete visitation sequences across trials. For both analyses, we used a first-order Markov model in which each dispenser was treated as a state and the probability of the next dispenser was estimated conditional on the current location. To isolate this organization independently of revisits or home returns, analyses were restricted to trials in which animals visited all eight peripheral dispensers exactly once. Identical procedures were applied to sequences generated by stochastic reference models, recomputed to select only among yet-unvisited dispensers, providing null expectations for predictive performance.

#### A first-order Markov model captures highly predictable local transitions

Local predictability was quantified by cross-validation, evaluating how accurately the next dispenser could be predicted from the current location using transition probabilities learned on a subset of trials and tested on held-out trials.

**Figure 5:**
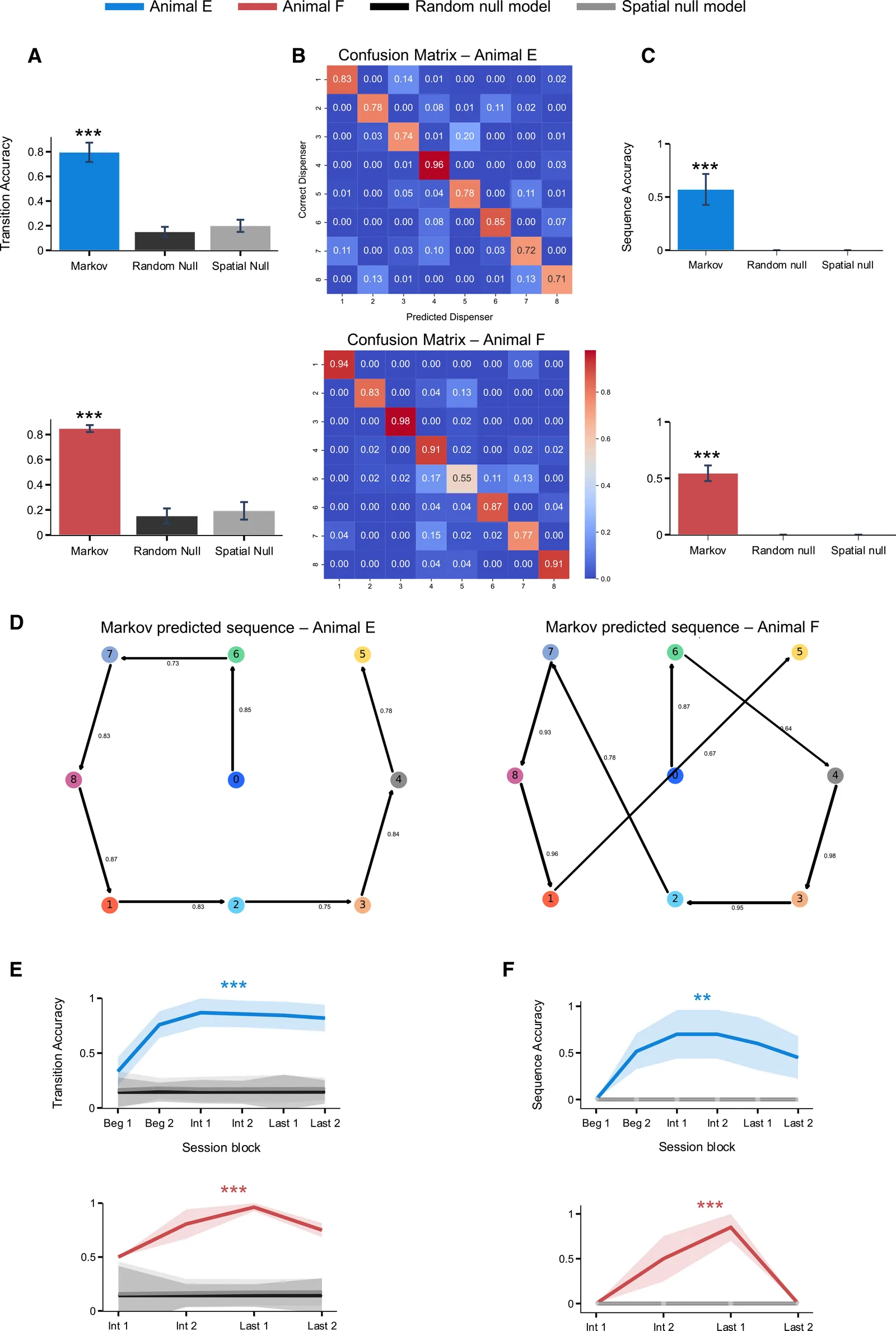
Spatial organization combines strong local predictability with sequence-level variability (A) Local transition prediction accuracy. Bar plots show mean accuracy of next-dispenser prediction using a first-order Markov model for Animal E (*top*) and Animal F (*bottom*). Accuracy was evaluated using cross-validation on trials without non-rewarded events; error bars indicate 95% confidence intervals across cross-validation folds. Performance of the empirical Markov model is compared to two stochastic reference models (uniform random-choice and spatially constrained random-walk models), recomputed to select only among yet-unvisited dispensers. Null distributions were generated by resampling sequences from the reference models separately for each animal, with the number of generated sequences matched to the number of empirical trials. Statistical significance was assessed using Monte Carlo tests with Bonferroni correction. (B) Confusion matrices for the Markov model. Confusion matrices show predicted versus actual next-dispenser choices for Animal E (*top*) and Animal F (*bottom*). Rows correspond to the true next dispenser and columns to the dispenser predicted by the model. Rows are normalized to show the distribution of predictions conditional on the true dispenser. Diagonal entries indicate correct predictions and illustrate the degree of local transition predictability within each block. (C) Sequence-level prediction accuracy. Bar plots show the accuracy of full visitation sequence prediction using the Markov model for Animal E (*top*) and Animal F (*bottom*). Sequence prediction accuracy reflects the proportion of trials for which the ordered sequence of dispenser visits was exactly predicted. Error bars indicate 95% confidence intervals across cross-validation folds. Reference model performance and statistical comparisons were computed using the same procedures as in A. (D) Markov-predicted visitation sequences. Predicted visitation sequences generated by the Markov model trained on all trials without non-rewarded events for Animal E (*left*) and Animal F (*right*), by iteratively selecting the most probable next transition from the learned Markov transition matrix. Nodes represent dispenser locations arranged according to their spatial positions in the environment. Directed edges indicate the sequence of predicted transitions, with edge thickness proportional to transition probability. Labels indicate transition probabilities. (E) Local transition predictability across session blocks. Line plots show the evolution of local transition prediction accuracy across session blocks for Animal E (*top*) and Animal F (*bottom*). Shaded regions indicate 95% confidence intervals across cross-validation folds. Black and gray lines indicate performance of the uniform random-choice and spatially constrained random-walk models, respectively, computed by resampling sequences separately within each session block and animal, with sequence counts matched to the empirical data. Differences between the Markov model and reference models were assessed using Monte Carlo tests with Bonferroni correction. Changes across session blocks were assessed using Kruskal-Wallis tests followed by Dunn post hoc comparisons with Bonferroni correction. Asterisks indicate significant differences across session blocks. (F) Sequence-level predictability across session blocks. Line plots show the evolution of full visitation sequence prediction accuracy across session blocks for Animal E (*top*) and Animal F (*bottom*). Shaded regions indicate 95% confidence intervals across cross-validation folds. Reference model performance and statistical comparisons were computed using the same procedures as in E. Significance levels: ∗*p* < 0.05; ∗∗*p* < 0.01; and ∗∗∗*p* < 0.001. See also Figure S5.

Across cross-validation folds, the Markov model showed strong predictive performance at the local level (Figures 5A and 5B), correctly predicting the next dispenser with average accuracies of 79.6 ± 7.8% for Animal E and 84.8 ± 2.7% for Animal F (mean ±95% CI). In both animals, this performance was far above that expected under either stochastic reference model (Monte Carlo tests, Bonferroni-corrected across models, all *p* < 0.001), indicating that the current location carried substantial information about the animal’s next choice.

These results indicate that navigation exhibits strong local regularities, with highly predictable dispenser-to-dispenser transitions.

#### Full visitation sequences remain variable despite strong local regularities

We next asked whether this local organization was sufficient to account for the ordering of dispenser visits across entire trials. To this end, we used Markov transition matrices to reconstruct full visitation sequences, starting from the empirically observed first dispenser and iteratively selecting the most probable next transition at each step (Figure 5D).

Sequence prediction accuracy was quantified as the proportion of test trials for which the entire predicted visitation sequence exactly matched the observed sequence (Figure 5C). Across cross-validation folds, sequence accuracy was lower than local transition accuracy but remained well above null expectations, reaching 57.1 ± 14.5% for Animal E and 54.5 ± 6.9% for Animal F (mean ±95% CI). In contrast, both stochastic reference models failed to reproduce any complete visitation sequence (0% accuracy; Monte Carlo tests, Bonferroni-corrected, all *p* < 0.001).

Thus, although individual dispenser-to-dispenser transitions were highly predictable, this local organization was not sufficient to uniquely specify complete visitation sequences.

#### Local and sequence-level predictability follow distinct trajectories across session blocks

We next examined how local and sequence-level predictability evolved with experience by quantifying the predictive performance of a first-order Markov model trained separately within each session block using the same cross-validated procedures as above. Trials without non-rewarded events first appeared in different blocks for the two animals (Animal E: Beg 1; Animal F: Int 1), but once present, both animals exhibited qualitatively similar trajectories across session blocks.

At the local level, the Markov model consistently outperformed both stochastic reference models in every session block (Figure 5E), indicating strong local transition predictability from the earliest analyzed blocks. Local prediction accuracy increased significantly across session blocks (Kruskal-Wallis tests; Animal E: *H* = 22.3, *p* < 0.001; Animal F: *H* = 16.7, *p* < 0.001) and was higher in later blocks (Dunn tests, Bonferroni-corrected, all *p* < 0.05; Figure S5A).

At the sequence level, predictability followed a markedly different trajectory (Figure 5F). In the earliest analyzed blocks, full visitation sequences were not predicted above chance (Monte Carlo tests, Bonferroni-corrected, all *p* > 0.1), indicating low reproducibility across trials. With experience, sequence prediction accuracy increased significantly in both animals (Kruskal-Wallis tests; Animal E: *H* = 17.8, *p* < 0.01; Animal F: *H* = 22.5, *p* < 0.001; Figure S5B), with intermediate blocks showing robust above-chance accuracy (Monte Carlo tests, Bonferroni-corrected, *p* < 0.001). However, this increase was not sustained: sequence prediction accuracy declined in later blocks (Dunn tests, Bonferroni-corrected, all *p* < 0.05), and for Animal F no longer exceeded null expectations (Monte Carlo tests, Bonferroni-corrected, *p* > 0.1).

Together, these results reveal a temporal dissociation between local and sequence-level organization across experience. While local predictability was expressed early and strengthened with experience, sequence-level predictability followed a non-monotonic trajectory, indicating that organization at the level of full visitation sequences remained flexible rather than converging onto a fixed pattern.

### A core set of transitions supports flexible recombination of full visitation sequences

In the previous section, we showed that navigation exhibited a scale-dependent organization, with highly predictable local transitions alongside substantial variability in full visitation sequences. To understand how these properties could coexist, we next analyzed complete visitation sequences, focusing on how they vary and relate to one another.

#### Complete sequences are variable but share a stable core set of transitions

**Figure 6:**
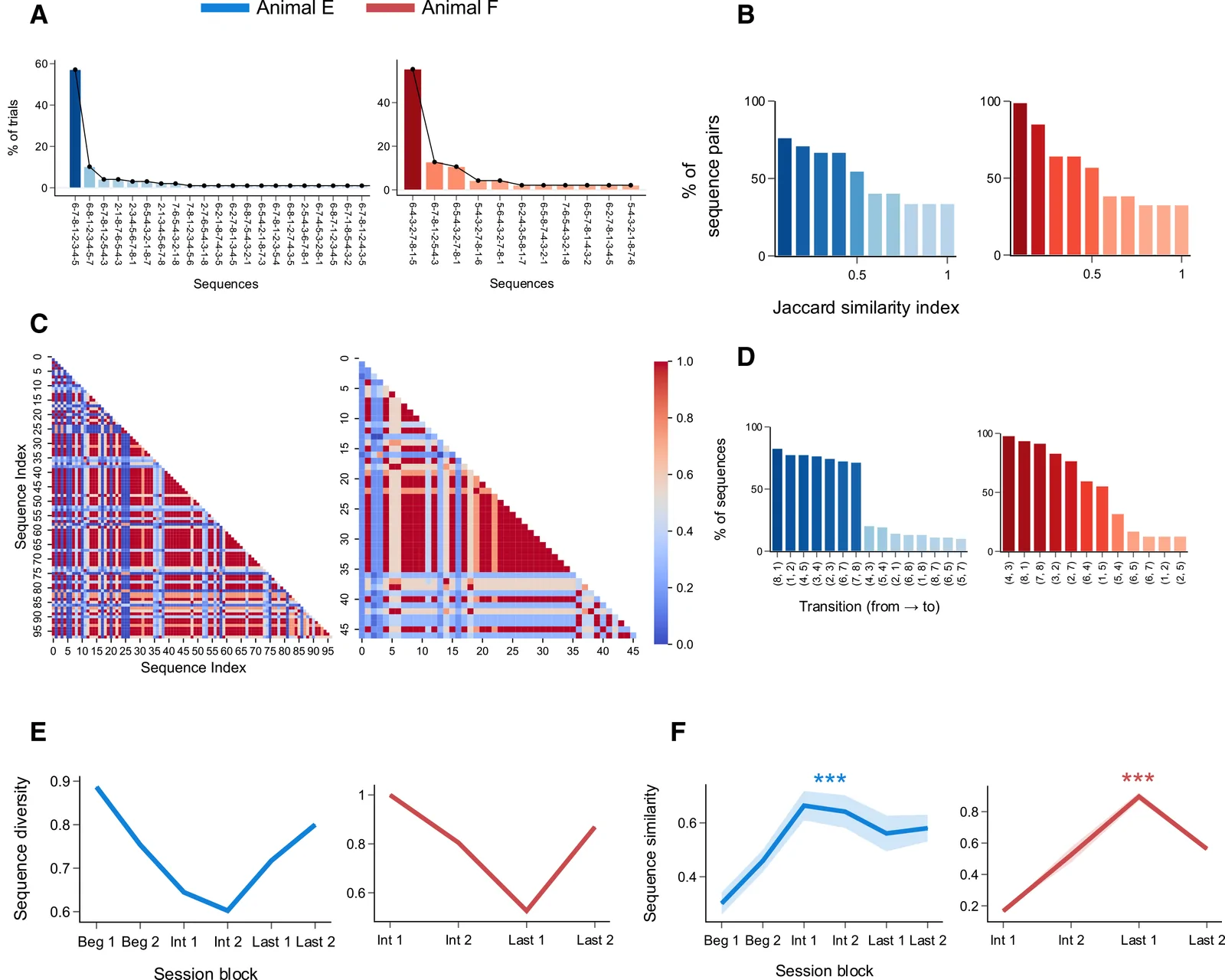
Flexible recombination of visitation sequences from a shared set of transitions (A) Distribution of complete visitation sequences. Bar plots show the percentage of trials in which each unique complete visitation sequence was observed, for trials without non-rewarded events, for Animal E (*left*) and Animal F (*right*). Sequences are ordered by decreasing percentage. (B) Overlap in transition content between visitation sequences. Histograms show the distribution of pairwise Jaccard similarity indices computed between all pairs of complete visitation sequences for Animal E (*left*) and Animal F (*right*). The Jaccard index quantifies the proportion of shared dispenser-to-dispenser transitions between two sequences (range: 0–1). (C) Pairwise similarity matrices between visitation sequences. Heatmaps show pairwise Jaccard similarity indices between all complete visitation sequences for Animal E (*left*) and Animal F (*right*), computed as in B. Each cell represents the similarity between a pair of sequences. (D) Recurrence of individual transitions across visitation sequences. Bar plots show the proportion of complete visitation sequences in which each dispenser-to-dispenser transition occurred for Animal E (*left*) and Animal F (*right*). Transitions are ordered by decreasing recurrence. (E) Evolution of sequence diversity across session blocks. Line plots show sequence diversity across session blocks for Animal E (*left*) and Animal F (*right*), quantified using a normalized Shannon diversity index (range: 0–1). Each point corresponds to a session block and reflects variability in complete visitation sequences expressed within that block. (F) Evolution of sequence similarity across session blocks. Line plots show mean pairwise Jaccard similarity between visitation sequences within each session block for Animal E (*left*) and Animal F (*right*). Shaded areas represent the SEM computed across sequence-level mean similarity values within each block. Changes across blocks were assessed using Kruskal-Wallis tests, followed by Dunn post hoc comparisons with Bonferroni correction. Asterisks indicate significant differences across session blocks (∗∗∗*p* < 0.001). See also Figure S6.

We first characterized the diversity and recurrence of complete visitation sequences expressed in trials without non-rewarded events (Figure 6A). In both animals, behavior was dominated by a single visitation sequence, which accounted for a large fraction of trials (Animal E: 57.1%; Animal F: 55.3%). Alongside this dominant sequence, animals expressed multiple alternative visitation orders (Animal E: 22 unique sequences; Animal F: 11), indicating substantial variability at the level of full visitation sequences.

To assess whether distinct visitation sequences shared common transition content, we decomposed each complete sequence into its constituent dispenser-to-dispenser transitions and quantified pairwise similarity using the Jaccard similarity index (Figures 6B and 6C; range: 0–1). Despite variability in visit order, sequences exhibited pronounced overlap in their transition content. Mean pairwise similarity (± SEM across sequence pairs) was 0.54 ± 0.01 in Animal E and 0.58 ± 0.01 in Animal F, with more than half of sequence pairs sharing at least 50% of their transitions in both animals.

This overlap was further reflected in the recurrence of a limited subset of transitions across most sequences (Figure 6D). In Animal E, seven transitions appeared in more than 70% of visitation sequences, while in Animal F, five transitions appeared in more than 75% of sequences.

Together, these results show that animals expressed multiple visitation sequences, but that these sequences were assembled from different combinations of a shared core set of frequently used transitions.

#### Sequence diversity evolves non-linearly across experience

We next examined how variability in complete visitation sequences evolved with experience. Sequence diversity within each session block was quantified using a normalized Shannon diversity index (Figure 6E; range: 0–1).

In both animals, sequence diversity followed a non-monotonic trajectory across session blocks, with higher diversity in early and late analyzed blocks and reduced diversity at intermediate stages. This pattern was accompanied by corresponding changes in the dominance of the most frequent visitation sequence (Figure S6A).

To characterize how sequence composition changed across experience, we distinguished between newly observed sequences (new sequence ratio; NSR) and sequences that had been expressed in earlier blocks (reused sequence ratio; RSR; Figures S6B and S6C). In the earliest session blocks, sequence variability was associated with a higher proportion of newly observed visitation sequences, whereas in later blocks variability primarily reflected the reuse of previously expressed sequences.

Together, these analyses show that sequence diversity changed non-linearly across experience, shifting from variability associated with newly expressed visitation sequences to variability dominated by reuse of previously established sequences.

#### Increasing overlap in transition content between visitation sequences over experience

Finally, we asked whether the relationships between visitation sequences changed with experience. Pairwise similarity between complete visitation sequences increased significantly across session blocks in both animals (Kruskal-Wallis; Animal E: *H* = 31.2, *p* < 0.001; Animal F: *H* = 33.4, *p* < 0.001; Figure 6F; Figure S6D), with intermediate and later session blocks showing significantly higher similarity values than the earliest analyzed blocks (Dunn tests, Bonferroni-corrected, all *p* < 0.01).

Importantly, this increase in similarity was not restricted to individual session blocks. Visitation sequences expressed in later blocks shared many transitions with sequences expressed earlier (Figure S6E), indicating that a substantial fraction of transition elements was preserved across experience rather than being specific to particular stages.

Together, these results show that overlap in transition content between visitation sequences increased with experience, indicating progressive convergence toward a shared set of transitions despite continued variability in visit order.

### Marmoset navigation exhibits a state-dependent multi-step predictive organization

The analyses above showed that navigation combined highly predictable local transitions with flexible ordering of full visitation sequences. This raised a key question: despite the variability of full sequences, did the current location nevertheless constrain the distribution of visits over multiple subsequent steps beyond what was captured by immediate transition probabilities? In other words, did the current location carry information about which dispensers were likely to be visited over the next several steps, independently of the exact order in which they would be visited? To address this, we used the successor representation (SR), a formalism that characterizes each state by its expected discounted future occupancy of all other states and thereby provides a principled description of state-dependent multi-step predictive organization.

#### Marmoset navigation exhibits state-dependent multi-step predictability

**Figure 7:**
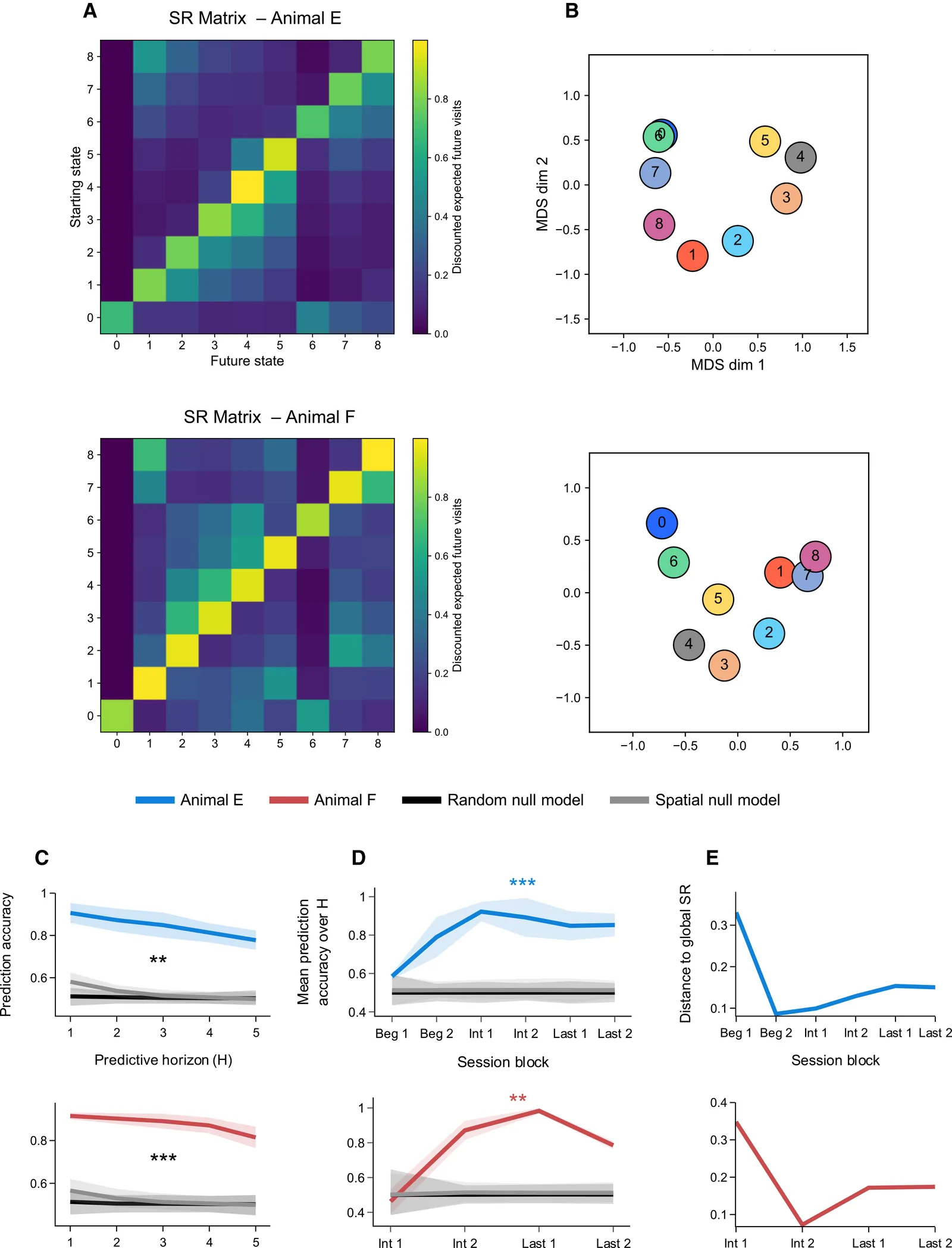
A state-dependent multi-step predictive organization in marmoset navigation (A) Successor representation matrices. Successor representation (SR) matrices for Animal E (*top*) and Animal F (*bottom*), computed from trials without non-rewarded events. Rows correspond to starting dispensers (states), and columns correspond to future dispensers. Color indicates the discounted expected future visits. (B) Low-dimensional embedding of predictive profiles. Classical multidimensional scaling (MDS) embeddings of the SR matrices for Animal E (*top*) and Animal F (*bottom*). Each point represents a dispenser location, and distances between points reflect similarity between their SR predictive profiles. (C) Multi-step predictive accuracy of the successor representation. Line plots show predictive accuracy (area under the ROC curve, AUC) of SR-based predictions as a function of predictive horizon (*H* = 1–5) for Animal E (*top*) and Animal F (*bottom*). Predictive accuracy reflects how well the SR, given the current dispenser, ranked yet-unvisited dispensers according to their likelihood of being visited within the next H steps. Shaded regions indicate 95% confidence intervals across cross-validation folds. Performance of the SR is compared to two stochastic reference models (uniform random-choice and spatially constrained random-walk models), recomputed to select only among yet-unvisited dispensers. Null distributions were generated by resampling sequences from the reference models separately for each animal, with the number of generated sequences matched to the number of empirical trials. Statistical significance was assessed using Monte Carlo tests with Bonferroni correction. (D) Emergence of multi-step predictive accuracy across session blocks. Line plots show the evolution of mean SR predictive accuracy across session blocks for Animal E (*top*) and Animal F (*bottom*), averaged across predictive horizons (*H* = 1–5). Shaded regions indicate 95% confidence intervals across cross-validation folds. Black and gray lines indicate performance of the uniform random-choice and spatially constrained random-walk reference models, respectively, shown here for visualization. Changes across session blocks were assessed using Kruskal-Wallis tests followed by Dunn post hoc comparisons with Bonferroni correction. Asterisks indicate significant effects across session blocks. (E) Convergence of block-specific successor representations. Line plots show the Frobenius distance between block-specific SR matrices and the global SR (computed across all analyzed blocks) for Animal E (*top*) and Animal F (*bottom*). Lower values indicate greater similarity between block-level and global multi-step predictive organization. Significance levels: ∗*p* < 0.05; ∗∗*p* < 0.01; and ∗∗∗*p* < 0.001. See also Figure S7.

We constructed SR matrices from trials without non-rewarded events, in which animals visited each peripheral dispenser exactly once. SRs were computed with discount factor *γ* = 0.7, selected by cross-validated predictive performance (Figure S7A). In the SR framework, γ determines how strongly more distant future states contribute to each state’s predictive profile. At this intermediate value, the SR integrates information over a moderate predictive horizon, spanning several future steps rather than being dominated by either the immediate next transition or the full remaining sequence. The resulting SR yields, for each dispenser, a predictive profile describing the expected distribution of future visits over subsequent steps, conditional on the current location (Figure 7A).

To visualize these predictive profiles, we embedded the SR matrices into a low-dimensional space using classical multidimensional scaling (MDS; Figure 7B), where distances between locations reflect similarity in future-occupancy distributions. Dispensers occupied non-uniform positions in this space, indicating heterogeneous predictive profiles across locations. Applying the same procedure to sequences generated by both reference models (uniform random-choice and spatially constrained random-walk; Figure S7B) produced markedly less separation between locations, indicating that the differentiation of predictive profiles across dispensers reflected state-dependent predictive organization rather than random sampling or spatial proximity alone.

We next tested whether this predictive organization was expressed in navigation behavior by evaluating multi-step predictive accuracy across horizons *H* = 1–5 (Figure 7C). For each held-out trial, we asked how well the SR, given the current dispenser, ranked yet-unvisited dispensers by their likelihood of being visited within the next H steps (area under the ROC curve; cross-validation). Across horizons, SR-based predictions achieved high predictive accuracy in both animals (Animal E: mean AUC = 0.84; Animal F: mean AUC = 0.88, both averaged across horizons *H* = 1–5) and consistently exceeded null expectations derived from both reference models (Monte Carlo tests, Bonferroni-corrected, all *p* < 0.01).

Together, these results show that future visits were not uniformly distributed from a given location, nor explained by spatial proximity alone. Instead, the current location predicted a non-uniform distribution of upcoming visits over multiple steps, consistent with a state-dependent multi-step predictive organization.

#### Multi-step predictive organization emerges and stabilizes with experience

We next examined how the state-dependent multi-step predictive organization captured by the SR evolved with experience. Specifically, we asked whether this organization emerged gradually across session blocks and whether it converged toward a consistent form over repeated interaction with the environment.

We computed SRs separately within each session block and assessed multi-step predictive accuracy on held-out trials from the same block across horizons *H* = 1–5 (Figure S7C). In the earliest analyzed blocks, SR performance did not exceed null expectations derived from stochastic reference models (Monte Carlo tests, Bonferroni-corrected, all *p* > 0.1). With experience, SR predictive accuracy became reliably above null expectations in intermediate and later blocks in both animals (all *p* < 0.01), indicating the emergence of reliable state-dependent multi-step predictability.

Mean predictive accuracy, averaged across prediction horizons *H* = 1 to 5 (Figure 7D), increased significantly across session blocks (Kruskal-Wallis; Animal E: *H* = 15.92, *p* < 0.001; Animal F: *H* = 14.93, *p* < 0.01), with post hoc comparisons confirming higher predictive performance in intermediate blocks compared to the earliest blocks (Dunn tests, Bonferroni-corrected, *p* < 0.05), indicating a progressive strengthening of state-dependent multi-step predictive organization with experience.

To assess whether the predictive organization itself became more consistent over experience, we quantified the distance between block-specific SRs and the global SR computed from all eligible trials pooled across sessions, using the Frobenius norm (Figure 7E). In both animals, distances were largest in the earliest analyzed blocks and decreased substantially thereafter, remaining low in subsequent blocks, consistent with increasing stabilization of multi-step predictive organization across session blocks.

Together, these analyses indicate that state-dependent multi-step predictive organization emerged progressively with experience and became increasingly consistent, in line with its increasingly stable expression in navigation behavior.

## Discussion

In the present study, we asked how spatial organization emerges through repeated experience in two freely moving common marmosets foraging in a semi-naturalistic depletion-based environment. With experience, animals increasingly aligned their behavior with the depletion contingency, more consistently selecting yet-unvisited locations. This behavioral change was largely accounted for by the progressive consolidation of a stable transition scaffold between food locations, alongside sensitivity to the current depletion state within each foraging episode. This scaffold organized navigation at multiple scales: local dispenser-to-dispenser transitions became increasingly reliable, whereas complete visitation sequences remained variable, consistent with flexible recombination of reliable local transitions. Beyond this, the current location predicted the distribution of future visits over multiple subsequent steps, revealing a state-dependent multi-step predictive organization at the behavioral level that stabilized progressively with experience. Together, these findings indicate that multi-scale spatial organization emerges with repeated foraging experience in freely moving marmosets.

### A semi-naturalistic depletion-based framework for studying navigation in freely moving marmosets

Many classical approaches used to study navigation in non-human primates (NHPs) have relied on touchscreens, simplified mazes, virtual environments, or otherwise constrained forms of locomotion. Although highly informative, these paradigms necessarily restrict important components of natural navigation, including vestibular, proprioceptive, and motor signals, as well as the continuous coupling between movement and sensory sampling. This limitation is especially relevant for the common marmoset, a small arboreal primate that has recently attracted growing interest as a model for spatial navigation. In the wild, marmosets navigate complex three-dimensional environments through visually guided movement,^12^^,^^13^ a form of spatial behavior that constrained laboratory paradigms are not well suited to capture. Freely moving paradigms are increasingly being developed in NHPs,^19^ including the marmoset, where they have revealed hippocampal spatial activity during free navigation.^20^^,^^21^ These studies, however, have focused on the neural correlates of navigation, without examining how spatial behavior changes with experience. Here, we instead took marmoset spatial behavior itself as the object of study, asking how it evolves as animals repeatedly forage in a semi-naturalistic environment, in which they could move freely in three dimensions and express their natural locomotor repertoire under controlled conditions. Such an approach aligns with the growing effort in neuroscience to design ethologically relevant paradigms known to reveal phenomena less accessible to more constrained settings.^1^^,^^2^^,^^22^^,^^23^^,^^24^

The ecological grounding of the paradigm derives, in particular, from the foraging problem it instantiates. The depletion contingency captures a challenge animals routinely face in nature, namely spatially distributed resources that cannot be profitably revisited once exploited, central to classic accounts of optimal foraging theory.^17^^,^^18^ Such designs provide a theoretically grounded framework in which behavior is shaped by the foraging contingency itself rather than by explicit training demands.^8^^,^^16^ This is particularly relevant for marmosets, which are natural foragers that exploit distributed resources.^10^^,^^12^^,^^13^ Although simplified relative to natural foraging, the paradigm preserved key features of an ecological foraging context, and the spatial patterns observed here are broadly consistent with field observations in marmosets.^14^^,^^25^ More broadly, foraging in NHPs has been studied from several angles: through its decision dynamics, such as how value, risk, and effort are weighed when choosing where to forage^26^; through tests of spatial memory for resource locations^27^^,^^28^; and, mostly in the field, through how primates plan and structure their trajectories among multiple food sites.^29^^,^^30^ This last line of work is closest to our own question, but has been conducted almost entirely in the wild, where the gradual emergence of spatial organization is difficult to track. We therefore took a complementary, longitudinal perspective, tracking how the spatial organization of foraging takes shape over time rather than at a single stage. This dynamic view captures how marmosets gradually structure their spatial behavior to exploit a familiar environment.

A defining feature of our minimally constrained paradigm is that marmosets were free both to choose where to go and how fully they completed each depletion episode. This generated substantial trial-to-trial heterogeneity, which clustering captured as distinct execution profiles reflecting different ways of interacting with the depletion contingency. Importantly, only the dominant complete-sampling profiles changed systematically with experience, whereas the less frequent incomplete profiles remained comparatively stable. This suggests that the dominant profiles were most closely linked to experience-dependent adaptation to depletion, whereas the others likely reflected additional sources of variability such as arousal, activity level, or sustained participation. This way of decomposing freely expressed behavior into distinct profiles reflects a central strategy in computational ethology for making naturalistic behavior tractable,^1^^,^^31^ which typically seeks to isolate qualitatively distinct behavioral states. Here, by contrast, these dominant profiles did not appear to be categorically distinct, but rather graded expressions of a shared transition scaffold. This continuity is what justified pooling them, shifting the focus of subsequent analyses to the shared scaffold underlying these profiles, how it consolidated with experience, and how it organized navigation across scales.

### Alignment with the depletion contingency reflects multiple components operating at different timescales

A central finding of the present study is that behavior became increasingly aligned with the depletion contingency over experience, as animals more consistently directed early choices toward dispensers that still contained reward. This alignment could not be explained by undirected sampling or by local spatial proximity alone, raising the question of which functional components supported it. A useful way to approach this question is to consider the different temporal scales intrinsic to the paradigm. Across session blocks, animals repeatedly interacted with the same stable spatial layout, creating conditions for the progressive consolidation of spatial regularities. This was reflected in the way transition structure evolved with experience: transition entropy decreased across session blocks, while transition matrices remained highly correlated from early experience and became progressively sharper in their relative weighting. This longer-timescale component is compatible with what has classically been described as spatial reference memory^15^ and is consistent with recent evidence that freely exploring marmosets retain spatially relevant information across repeated exposures.^28^

At the same time, the state of the environment changed dynamically within each trial, as each newly visited dispenser became depleted. Choice outcomes depended not only on transition structure, but also on the animal’s position within the unfolding depletion episode. This is demonstrated by the structure-only agent, which relied on transition probabilities alone without tracking within-trial visit history. Although this agent captured a substantial fraction of empirical behavior, it nonetheless underestimated reward probability and overall reward rate, indicating an additional contribution from within-trial visit history. Functionally, this shorter-timescale component is compatible with a working-memory contribution to spatial choice,^16^ consistent with evidence that marmosets retain information over short delays in working-memory tasks^32^ and use spatial memory to avoid revisiting depleted locations.^27^ However, the present results do not isolate working memory specifically, and other short-timescale processes that reduce returns to recently visited locations could also contribute.

Together, these findings suggest that alignment with the depletion contingency depended on both a longer-timescale transition structure that sharpened across sessions and a shorter-timescale component sensitive to the current depletion state within each foraging episode. This pattern resonates with field observations that marmosets employ both short- and long-term spatial memory in naturalistic foraging contexts,^10^ without implying the stronger claim that the present results isolate discrete mnemonic systems.

### Transition structure organizes navigation across multiple behavioral scales

The consolidation of a transition scaffold with experience raised a broader question: what kind of spatial organization emerged from it, and how does it relate to existing accounts of spatial navigation? To address this, we examined navigation at three scales: immediate dispenser-to-dispenser transitions, complete foraging sequences, and predictive relations between dispensers over multiple future steps. We therefore used computational models with well-characterized relational properties as analytical tools to characterize predictive organization at the behavioral level, without implying that marmosets actually implement these computations or that a specific cognitive or neural mechanism is thereby demonstrated.

At the scale of individual dispenser-to-dispenser transitions, a first-order Markov model captured local transition regularities by quantifying how strongly the current dispenser constrained the next one. Its high predictive performance suggests that navigation came to rely on increasingly reliable local transitions, beyond what could be explained by undirected sampling or local proximity alone. One plausible interpretation is that repeated experience progressively reinforced specific rewarded transitions, thereby sharpening local transition preferences. However, strong local predictability does not by itself determine the larger-scale organization that emerges. Reliable local transitions could in principle support either rigid, route-like behavior or more flexible navigation, a contrast long recognized in reinforcement-learning (RL) accounts of behavior.^33^^,^^34^ Distinguishing these possibilities therefore requires examining navigation behavior at larger scales.

A classical route-based strategy^4^^,^^7^ would predict that animals assemble local transitions into fixed global routes. Behaviorally, this implies that complete visitation sequences should become as reproducible as the individual transitions composing them: if navigation followed a stereotyped route, knowing the current dispenser would predict not only the next one but the entire remaining sequence. Our data did not match this prediction. Sequence-level predictability remained well below local predictability and did not converge toward a single stable pattern, as multiple alternative visitation sequences continued to be expressed throughout experience. Local transition regularities therefore did not crystallize into rigid route replay but instead supported a more flexible ordering of sequences. This flexibility was evident in the dynamics of complete sequences. Sequence diversity followed a non-monotonic trajectory across experience, while distinct sequences showed increasing overlap in the transitions they contained. Together, this indicates that experience stabilized a shared repertoire of transitions that could be recombined in different orders.

Whether this flexibility was itself constrained could not be resolved from sequence ordering alone: it required asking whether the current dispenser predicts which dispensers will be visited over several subsequent steps, regardless of their exact order. We addressed this using the SR, an RL formalism that characterizes each dispenser by its expected future occupancy of the others over several steps.^35^^,^^36^ The current dispenser predicted upcoming visits well above stochastic reference models, and this multi-step predictive organization emerged and stabilized with experience. Notably, it remained stable even as the reproducibility of complete sequences rose and fell across experience: the renewed variability of later sessions stayed bounded by the same multi-step relations between dispensers. Behavioral flexibility therefore appeared to operate within a stable relational organization, in which the dispensers likely to be reached over the next few steps were predictable, even when the exact order of their visits was not.

Our findings relate marmoset foraging to established accounts of spatial navigation. SR-like organization has previously been identified in rats and humans.^37^^,^^38^ Whereas these studies fitted RL agents to behavior as generative models of choice, we used the SR descriptively to ask whether predictive organization was present in freely expressed foraging and how it emerged with experience. Consistent with this, marmosets’ behavior came to contain the kind of multi-step relational organization the SR formalizes. Such a relational organization can be understood in terms of graph-like or topological accounts of navigation, in which space is organized as a set of connections between places, that is, which places can be reached from which.^3^^,^^6^ This is consistent with field observations in wild marmosets, whose foraging combines reused routes with flexible adjustments within what has been interpreted as a topological map.^25^ The convergence of this pattern across such different contexts, free-ranging foraging in a semiarid habitat and controlled foraging in captivity, raises the possibility that this may be a recurring feature of marmoset spatial behavior.

### Neural and comparative perspectives

The present findings are broadly compatible with the involvement of partially distinct neural systems. The dissociation between a longer-timescale transition scaffold and a shorter-timescale component sensitive to within-trial depletion state is consistent with complementary contributions of hippocampal, prefrontal, and striatal systems to spatial behavior.^5^^,^^26^^,^^34^^,^^39^^,^^40^^,^^41^ Within this broader framework, the multi-step predictive organization identified here at the behavioral level raises the possibility that future neural studies in freely moving marmosets may reveal representations consistent with predictive-map accounts of hippocampal function.^42^ Such studies may be especially informative in the common marmoset, where hippocampal activity during natural navigation appears to be strongly shaped by visual exploration and three-dimensional space.^20^

Beyond these neural considerations, the present findings invite broader comparison. In wild NHPs, spatial foraging has often been described in topological terms,^9^ organized around networks of familiar routes between key locations. Our results illustrate how such a relational organization, combining reliable local structure with larger-scale flexibility, takes shape with experience. A similar articulation of local and larger-scale structure has been described in humans, where navigation in unfamiliar environments relies on an adaptive combination of local transition information and larger-scale, landmark-based guidance.^43^ Whether this reflects similar organizational principles in marmosets and humans remains an open question, particularly given evidence that marmosets use landmark information to guide navigation but may do so differently from human adults.^44^ Finally, the progressive stabilization of predictive relations between locations without convergence toward a fixed route shares some features with accounts of spatial schema formation,^45^ and leaves open whether related forms of generalization might also be observed across environments in marmosets.

### Limitations of the study

While our paradigm enabled a rare longitudinal view of navigation in freely moving marmosets, a key limitation of the study is the small number of animals. Such constraints are common in primate neuroscience, where methodological and ethical considerations often restrict sample size.^46^^,^^47^ Accordingly, the present findings are best viewed as establishing a proof-of-concept behavioral and analytical framework in freely moving marmosets, rather than supporting broad generalization about primate navigation. Importantly, several core behavioral features were remarkably consistent across the two animals, strengthening confidence in the main patterns identified. The differences that remained concerned the precise expression and temporal dynamics of these shared principles and may reflect meaningful individual differences, consistent with evidence that stable differences in marmoset cognition are linked to personality-related traits and social environment.^48^ Moreover, both animals were male, and we could therefore not assess whether sex influences the spatial organization described here. A further limitation is that behavior was characterized in a single, fixed spatial layout. Because the environment did not vary, we cannot determine whether the organization described here would generalize to other spatial configurations or reflects features specific to this particular arrangement. Testing whether comparable organization emerges across different layouts is an important direction for future work. Finally, our study is purely behavioral, which limits mechanistic inference about the underlying neural substrates, although the findings are compatible with broader theoretical frameworks of spatial cognition. Future studies combining this paradigm with wireless neural recordings in freely moving marmosets will therefore be important for characterizing the circuit-level substrates of experience-dependent spatial organization during naturalistic foraging.

In summary, the present study establishes a semi-naturalistic depletion-based paradigm for investigating spatial navigation during self-guided foraging in freely moving common marmosets. Within this framework, repeated foraging experience was associated with the emergence of a multi-scale spatial organization in which a stable structure supported flexible behavior: increasingly reliable local transitions and stable multi-step predictive relations between locations coexisted with flexibly recombined visitation sequences. These findings identify behavioral features compatible with theoretical and computational accounts of spatial cognition and provide a tractable semi-naturalistic behavioral and analytical framework for future studies linking foraging behavior to neural activity in freely moving marmosets.

## Resource availability

### Lead contact

Requests for further information and resources should be directed to and will be fulfilled by the lead contact, Nada El Mahmoudi (nada.elmahmoudi@mail.mcgill.ca).

### Materials availability

This study did not generate new unique reagents.

### Data and code availability

• Data: all behavioral data reported in this paper have been deposited at Mendeley Data and are publicly available as of the date of publication. The DOI is listed in the key resources table.
• Code: All original code has been deposited at Zenodo and is publicly available as of the date of publication. The DOI is listed in the key resources table.
• All other items: any additional information required to reanalyze the data reported in this paper is available from the lead contact upon request.

## Acknowledgments

We thank the animal care staff at the Ernst Strüngmann Institute for their assistance in ensuring the welfare of the marmosets. Specific thanks to Colleen Illing for supporting the marmosets’ training. This work was supported by the 10.13039/501100002347Bundesministerium für Bildung und Forschung (ERANET 10.13039/100031366NEURON 01EW2110).

## Author contributions

Conceptualization: N.E.M., F.L., and J.L. Methodology: N.E.M. and F.L. Investigation: N.E.M. and F.L. Formal analysis: N.E.M. Resources: F.Z.N. Technical support: F.Z.N. and D.S. Writing - original draft: N.E.M. Writing - review and editing: N.E.M., F.L., and J.L. Funding acquisition: J.L. Supervision: J.L. All authors read and approved the final manuscript.

## Declaration of interests

The authors declare no competing financial or non-financial interests related to this work.

## Declaration of generative AI and AI-assisted technologies in the writing process

During the preparation of this work the authors used ChatGPT (OpenAI) to improve readability and language. After using this tool, the authors reviewed and edited the content as needed and take full responsibility for the content of the published article.

## STAR★Methods

### Key resources table

**Table:** 

REAGENT or RESOURCE	SOURCE	IDENTIFIER
**Deposited data**		

Raw behavioral data	This paper	Mendeley Data: https://doi.org/10.17632/769d7jwchw.1

**Experimental models: Organisms/strains**		

Common marmoset, *Callithrix jacchus* (adult males)	German Primate Center, Göttingen, Germany	N/A

**Software and algorithms**		

BORIS	Friard, O. and Gamba, M.^49^	https://www.boris.unito.it
Python (v3.12.5)	Python Software Foundation	https://www.python.org
NumPy (v1.26.4)	Harris.et al.^50^	https://numpy.org
pandas (v2.2.2)	McKinney^51^	https://pandas.pydata.org
statsmodels (v0.14.2)	Seabold, S. and Perktold, J.^52^	https://www.statsmodels.org
scikit-posthocs (v0.9.0)	Terpilowski^53^	https://github.com/maximtrp/scikit-posthocs
SciPy (v1.13.1)	Virtanen^54^	https://scipy.org
scikit-learn (v1.8.0)	pedregosa^55^	https://scikit-learn.org
NetworkX (v3.3)	Hagberg^56^	https://networkx.org
Plotly (v5.24.1)	Plotly Technologies Inc.	https://plotly.com/python
Original code	This paper	Zenodo: https://doi.org/10.5281/zenodo.21724716

### Experimental model and study participant details

#### Ethics statement

All animal procedures were approved by the Regierungspräsidium Darmstadt (permit F149/1012) and complied with all applicable German and European regulations governing animal husbandry and welfare. Given the intensive longitudinal design of the study, the experimental approach relied on extensive repeated sampling within each subject to enable detailed within-animal characterization while minimizing animal use and maintaining methodological rigor.^47^

#### Animals and housing

Two adult male common marmosets (*Callithrix jacchus*; mean age: 3 years) participated in the study. Animals were obtained from the German Primate Center (Göttingen, Germany) and pair-housed in a cage measuring 180 cm (H) × 90 cm (W) × 60 cm (D) under a 12 h light/dark cycle (lights on from 07:00 to 19:00). Room temperature was maintained at 24-25°C with approximately 60% relative humidity. Animals received two daily meals, approximately at 09:00 and 15:00, consisting of pellets, a dietary supplement (quark, yogurt, banana, and vitamins), and fresh vegetables. Water was available *ad libitum.* Gum arabic was provided exclusively during experimental sessions and was not part of the standard daily diet.

### Method details

#### Semi-naturalistic foraging arena

Experiments were conducted in a wire-mesh enclosure measuring 1.8 m × 1.4 m × 1.6 m (total volume ≈ 4.0 m^3^), allowing unrestricted three-dimensional locomotion. The mesh walls supported climbing and vertical movement. Access to the enclosure was provided via a tunnel system connecting a transport cage to one corner of the enclosure. To encourage exploration and locomotion, the enclosure was enriched with wooden sticks, semi-rigid ropes, platforms, and suspended enrichment elements. The enclosure was surrounded by four white walls decorated with landscape posters. Additional distal visual cues were provided by four colored curtains and large artificial plants positioned around the setup (Figure 1A).

#### Food dispensers and reward delivery

Rewards were delivered using custom-made, 3D-printed, remotely controlled dispensers (Figure 1E). Each dispenser was calibrated prior to each session to ensure accurate delivery of liquid gum arabic. Upon activation, each peripheral dispenser delivered approximately 0.15 mL of liquid gum arabic per trial. The reward was concealed within the dispenser spout and was not visible prior to retrieval. A central “home” dispenser (HD) was mounted upside down at the center of the ceiling. Activation of the HD triggered reward delivery along with concurrent visual (blue LED) and auditory (buzzer) cues.

#### Habituation

Prior to behavioral testing, animals underwent a multi-stage habituation protocol to ensure low anxiety and natural exploratory behavior in the enclosure. Animals were first habituated to transport from the animal facility to the laboratory. They were then introduced together into the enclosure, where they freely explored the environment and retrieved hidden food rewards placed at multiple locations.

In a subsequent phase, animals entered the enclosure individually while the partner remained in the transport cage nearby, maintaining visual contact. The duration of separation was gradually increased until each animal could remain alone in the enclosure for at least 30 minutes without signs of discomfort. Welfare was continuously monitored based on vocalizations and behavior, following established ethological criteria for captive marmosets.^13^

During habituation, animals were familiarized with the reward delivery mechanism using the same liquid gum arabic and dispenser interface used in the experiment. This familiarization was conducted in the housing facility. Consumption was monitored to verify that animals reliably retrieved the full gum arabic aliquot upon first contact, establishing that revisits during experimental sessions could not be attributed to incomplete consumption or residual reward availability.

#### Home dispenser training

To standardize trial initiation, animals were trained to voluntarily move to the center of the enclosure at the start of each trial. Training focused on associating activation of the home dispenser (blue LED and buzzer) with reward availability. Using positive reinforcement, animals learned to reach the HD promptly following cue onset.

At the beginning of each training trial, the HD was activated for approximately 3 seconds. The animal had up to 60 seconds to reach the HD for the trial to be considered successful. Trials were marked unsuccessful if the animal failed to reach the HD within this time window. Sessions were terminated if the animal failed two consecutive trials.

Training performance was quantified by the proportion of successful trials per session and the latency to reach the HD (interval between cue onset and the time the animal reached the HD). Training was considered complete once the animal achieved >80% successful trials in two consecutive sessions. Animal E reached criterion after 10 sessions, and Animal F after 18 sessions.

#### Depletion-based spatial foraging paradigm

Following home dispenser training, animals performed a depletion-based spatial foraging paradigm in which eight additional dispensers were installed at fixed locations along the enclosure perimeter to ensure broad spatial coverage (Figure 1A). Three dispensers were placed equidistantly along each of the two long sides of the enclosure, and one dispenser was placed at the midpoint of each short side. Dispenser positions remained fixed across all sessions.

Each trial constituted a depletion episode. Trials were initiated by activation of the HD, prompting the animal to move to the center of the enclosure within 60 seconds. Upon reaching the HD, all eight peripheral dispensers were activated simultaneously (Figure 1B), each releasing approximately 0.15 mL of gum arabic. Each dispenser delivered a single reward per trial and remained inactive thereafter until the next trial. Rewards remained available until collected.

To complete a trial, the animal was required to visit all eight peripheral dispensers at least once. The trial ended upon the first visit to the eighth unique peripheral dispenser, regardless of any revisits. If all eight dispensers were not visited within 10 minutes, the trial was terminated automatically. Upon trial completion or timeout, the HD was reactivated to signal the start of the next trial.

#### Session schedule and motivation

Each animal completed 30 experimental sessions. Sessions were conducted on separate days, typically spaced by approximately 24 h. Inter-session intervals were longer over weekends and, on rare occasions, exceeded 72 h due to logistical constraints.

Sessions took place between the animals’ two daily meals, during a period of natural foraging motivation. Participation was voluntary, and animals were free to disengage from the paradigm at any time. Sessions were terminated if the animal failed to initiate two consecutive trials (i.e., did not reach the home dispenser within 60 s). Animals completed 30 sessions each, yielding a total of 233 trials for Animal E and 232 trials for Animal F.

#### Behavioral event detection

All sessions were conducted by one experimenter at a time, with two experimenters alternating across sessions. The experimenter was positioned at a fixed location outside the enclosure. Dispenser visits and visit latencies were manually logged in real time using BORIS behavioral event-logging software.^49^

A visit to a dispenser was defined as the animal pausing in front of the dispenser and actively attempting to retrieve reward (e.g., reaching toward or licking the dispenser spout). This criterion ensured that only purposeful interactions with the dispenser were recorded as visits. Repeated contacts with the same dispenser during a single uninterrupted interaction bout were scored as a single visit; a new visit was counted only after the animal disengaged and subsequently returned.

All sessions were continuously recorded using a wide-angle camera mounted above the enclosure. Video recordings were systematically reviewed for all sessions to verify visit identity and ordering, and to resolve any discrepancies with the real-time event log. In all cases of discrepancy, the video recording was used as the authoritative source. For each trial, the ordered sequence of dispenser visits was extracted from the visit log.

### Quantification and statistical analysis

All analyses were performed in Python using standard scientific computing libraries, including NumPy, SciPy, pandas, scikit-learn, and statsmodels. Analyses were conducted separately for each animal. Unless otherwise specified, statistical tests were two-sided and significance was set at α = 0.05. When applicable, additional significance thresholds (*p* < 0.01 and *p* < 0.001) are reported. Exact sample sizes and units of analysis are reported below and in Table S4, which also summarizes the statistical tests and multiple-comparison procedures. Measures of central tendency and uncertainty, where applicable, are defined in the corresponding figure legends, and statistical results are reported in the Results and figure legends.

#### Spatial sampling of the foraging environment

To assess how animals sampled the spatial layout of the arena, we quantified interactions with the eight peripheral dispensers across all trials within each animal. Two complementary measures were considered: (i) the total number of visits to each dispenser, pooled across all trials and sessions, and (ii) the number of omissions per dispenser, defined as trials in which a given dispenser was never visited (trials terminated by timeout).

For each measure, observed distributions were compared to a uniform expected distribution using Chi-square goodness-of-fit tests. When significant deviations from uniformity were detected, post hoc two-sided binomial tests were performed for each dispenser to identify individual locations contributing to the effect. Bonferroni correction was applied to control for multiple comparisons across dispensers.

#### Non-rewarded events

##### Definition and quantification

To characterize how animals interacted with the depletion contingency, we focused on non-rewarded events, defined as behavioral outcomes within a trial that did not result in reward delivery. Three categories were distinguished: **revisits**, defined as visits to a peripheral dispenser already visited earlier within the same trial; **omissions**, defined as peripheral dispensers not visited within the trial, resulting in termination by timeout; and **home returns**, defined as visits to the central home dispenser occurring before all eight peripheral dispensers had been visited at least once.

For each trial, the number of events in each category was computed, along with the total number of non-rewarded events (sum across categories). Trial-level measures were then summarized at the session level as the mean number of events per session for each animal.

##### Temporal dynamics across sessions

Experience-dependent changes in non-rewarded events were assessed separately for each animal using linear regression on session-level mean event counts as a function of session number. Model fit was evaluated using R^2^, and 95% confidence intervals were computed for predicted values. Standard regression assumptions (linearity, independence of residuals, homoscedasticity, and normality of residuals) were assessed. Statistical inference was based on heteroscedasticity-consistent HC3 robust standard errors.

To assess the contribution of additional session-level covariates, multiple linear regression analyses were performed including session number, number of trials per session, time elapsed since the previous session (in days), and experimenter identity as predictors. Model assumptions were verified, multicollinearity was assessed, and statistical inference relied on robust standard errors. Correction for multiple hypothesis testing across predictors was performed using Bonferroni procedures.

##### Distribution of categories

To compare the relative prevalence of non-rewarded event categories, the frequencies of omissions, revisits, and home returns were compared at both the session and trial levels. Non-parametric Kruskal-Wallis tests were used to assess differences between event categories. When significant effects were detected, pairwise comparisons were performed using Dunn’s post hoc tests with Bonferroni correction. Session-level analyses were based on mean event counts per session, while trial-level analyses used individual trial values pooled across sessions.

##### Covariation with sampling behavior

To examine associations between non-rewarded events and overall sampling behavior, we computed Pearson correlation matrices including total non-rewarded events, event subcategories, and total number of visits. Correlation analyses were performed separately at the session level (using session averages) and at the trial level (using individual trials). *p*-values were corrected for multiple comparisons using Bonferroni correction.

#### Trial-level clustering analysis

##### Feature selection and preprocessing

To identify reproducible patterns of trial execution, clustering analyses were performed separately for each animal at the trial level using two complementary features: the total number of peripheral dispenser visits and the number of omissions. These features capture the extent and completeness of spatial sampling within a trial. Prior to clustering, features were standardized (z-scored) to ensure comparable scaling.

##### Clustering procedure

Clustering was performed using the k-means algorithm. Candidate solutions ranged from k = 2 to k = 6 clusters. The optimal number of clusters was selected by jointly considering within-cluster dispersion (inertia) and silhouette score across candidate solutions. Clustering was run with 100 random initializations to reduce sensitivity to initialization, and the solution maximizing silhouette score while minimizing inertia was retained. Trials were assigned to clusters based on proximity to cluster centroids in feature space.

##### Behavioral characterization

To characterize behavioral differences between clusters, we quantified multiple trial-level measures, including trial duration, total number of visits, total non-rewarded events, and non-rewarded event subcategories (revisits, omissions, and home returns). Distributions were compared across clusters by pooling trials belonging to each cluster. Statistical comparisons were conducted using Kruskal-Wallis tests. When significant effects were detected, pairwise comparisons were performed using Dunn’s post hoc tests with Bonferroni correction.

##### Evolution across sessions

To assess experience-dependent changes in cluster prevalence, the proportion of trials assigned to each cluster was computed for each session separately for each animal. Session-wise cluster proportions were analyzed as continuous summary measures, and linear regression was used to quantify changes in cluster prevalence as a function of session number. Standard regression assumptions were assessed, and statistical inference was based on heteroscedasticity-consistent HC3 robust standard errors.

#### Standardization of choice sequences

Each trial was represented as an ordered sequence of visits to the home dispenser and the eight peripheral dispensers. A choice was defined as each successive dispenser visit following the initial home visit, and a transition corresponded to the ordered pair formed by the current and subsequent dispenser. Because at most eight rewarded peripheral choices can occur within a trial, all transition-, sequence-, and model-based analyses described below were standardized to the first eight choices following the initial home visit. This window corresponds to the maximal number of potentially rewarded peripheral visits within a trial.

#### Definition of session blocks

To quantify experience-dependent changes while maintaining sufficient sample sizes within each partition, sessions were grouped into six consecutive five-session blocks: Beg 1 (sessions 1-5), Beg 2 (6-10), Int 1 (11-15), Int 2 (16-20), Last 1 (21-25), and Last 2 (26-30). Block-level analyses pooled trials belonging to the relevant behavioral subset (e.g., Clusters 0 and 1, or trials without non-rewarded events) within each block. Trials in Clusters 0 and 1 totaled 209 for Animal E and 208 for Animal F, with on average 34.8 ± 2.9 and 34.7 ± 3.9 trials per session block, respectively (mean ± SEM across six blocks).

#### Trials without non-rewarded events

For analyses requiring complete depletion episodes without non-rewarded events (sequence diversity, sequence similarity, Markov-model, and successor-representation analyses), we restricted the dataset to trials in which animals visited all eight peripheral dispensers exactly once, with no revisits, no omissions (i.e., no timeout), and no home returns before completion. This yielded 98 eligible trials for Animal E across all six blocks and 47 for Animal F across the four blocks containing eligible trials, corresponding to 16.3 ± 2.1 and 11.8 ± 3.1 eligible trials per analyzed block, respectively (mean ± SEM).

#### Stochastic reference models

To assess whether the observed behavioral patterns could arise from stochastic choice alone, empirical results were compared to reference models that generated dispenser selections according to predefined probabilistic rules while preserving the structural constraints of the trials (initial home visit, number of dispensers, and eight-choice window). Two reference models were considered: a uniform random-choice model and a spatially constrained random-walk model. Each model was implemented in two variants: one allowing non-rewarded events, used for empirical analyses in which revisits and home returns were permitted, and one disallowing non-rewarded events, used for analyses restricted to complete rewarded sequences.

##### Uniform random-choice model

In the uniform random-choice model, dispenser selections were generated by uniform sampling over the set of admissible dispensers at each step:

$$P\left(X_{t+1}=j\right)=\left\{ \begin{array}{cc} \frac{1}{|A_{t}|}, & \text{if}\;j\in A_{t}\\ 0, & \text{otherwise} \end{array}\right.$$

where *A*_*t*_ denotes the set of dispensers admissible for the choice following time step *t*. Simulated trials always started at the home dispenser and consisted of eight successive post-home choices. In the variant allowing non-rewarded events, all nine dispensers were admissible at every step (*A*_*t*_={0,…,8}). In the variant disallowing non-rewarded events, *A*_*t*_ consisted of the yet-unvisited peripheral dispensers, such that each simulated trial corresponded to a random permutation of the eight peripheral dispensers following the initial home visit.

##### Spatially constrained random-walk model

In the spatially constrained random-walk model, transitions between dispensers were biased by the geometric layout of the environment. Pairwise Euclidean distances *d*_*ij*_ between dispenser coordinates were computed in a fixed two-dimensional reference frame defined relative to the enclosure floor plan. Transition probabilities were defined as

$$P\left(X_{t+1}=j|X_{t}=i\right)=\left\{ \begin{array}{cc} \frac{d_{ij}^{-1}}{\sum_{k\in A_{t}}d_{ik}^{-1}}, & \text{if}\;j\in A_{t}\\ 0, & \text{otherwise} \end{array}\right.$$

where *A*_*t*_ denotes the set of dispensers admissible for the choice following time step *t*. For self-transitions, distances were set to the minimum non-zero inter-dispenser distance to avoid singularities in the inverse-distance kernel. Simulated trials started at the home dispenser and consisted of eight successive post-home choices. In the variant allowing non-rewarded events, all nine dispensers were admissible at every step (*A*_*t*_={0,…,8}). In the variant disallowing non-rewarded events, *A*_*t*_ consisted of the yet-unvisited peripheral dispensers, such that transition probabilities were renormalized at each step over that subset according to the same inverse-distance weighting.

##### Simulation procedure

For each reference model and variant, 5,000 independent simulation runs were generated, each comprising 500 trials. Each simulated trial started at the home dispenser and consisted of eight successive post-home choices. Simulated trials were stored and used as null pools for subsequent matched-N comparisons.

##### Monte Carlo inference and matched resampling

For comparisons between empirical data and stochastic reference models, we used matched-N Monte Carlo resampling. For each empirical partition (e.g., full dataset, cluster, or session block), the empirical statistic was computed across all included trials.

From the corresponding null pool, the same number of trials as in the empirical dataset was resampled with replacement within each simulation run, and the statistic was recomputed. Repeating this procedure across simulation runs yielded a null distribution matched in sample size to the empirical estimate. Null distributions were summarized using non-parametric 95% confidence intervals defined by the 2.5th and 97.5th percentiles of the matched null distribution.

For comparisons with stochastic reference models, one-sided Monte Carlo tests were applied in the direction of the *a priori* predicted deviation from null expectations (greater or lower, depending on the statistic). Monte Carlo *p*-values were computed using a +1 correction to avoid zero-valued estimates. When multiple comparisons were performed, *p*-values were corrected using Bonferroni correction.

#### Transition- and sequence-based analysis

##### Reward rate

Reward rate was defined for each trial as the proportion of rewarded choices (i.e., visits to yet-unvisited peripheral dispensers) within the standardized eight-choice window. Reward rate was compared across behavioral clusters and session blocks using non-parametric statistical tests (Mann-Whitney U for two-group comparisons or Kruskal-Wallis tests for multi-group comparisons, followed when appropriate by Dunn’s post hoc tests with Bonferroni correction). Comparisons to reference stochastic models were performed using matched-N Monte Carlo resampling procedures, as described above.

##### Global transition entropy

Global transition entropy was computed from the distribution of directed dispenser-to-dispenser transitions observed within the standardized eight-choice window. For a given set of trials, transitions between locations were pooled to obtain transition counts *C*_*ij*_ between locations *i* and *j*, which were normalized to yield a probability distribution over transitions:

$$p_{ij}=\frac{C_{ij}}{\sum_{m,n}C_{mn}}\text{.}$$

Global transition entropy was then computed as Shannon entropy (base 2) of this distribution:

$$H_{\text{global}}=-\sum_{i,j}\;p_{ij}\;\log_{2}\left(p_{ij}\right)\text{.}$$

Entropy was computed separately for each behavioral partition (clusters and session blocks). Uncertainty on entropy estimates was assessed using non-parametric bootstrap resampling at the trial level (1,000 resamples with replacement), with transition distributions recomputed after each resampling. Confidence intervals were obtained from the 2.5th and 97.5th percentiles of the bootstrap distribution.

Differences in global transition entropy between behavioral clusters were assessed using permutation tests (5,000 permutations) on entropy differences. For each comparison, trial sequences were pooled across groups and randomly reassigned while preserving the number of trials per group. Entropy was recomputed after each permutation, yielding a permutation-based null distribution of entropy differences under random group assignment. Two-sided empirical *p*-values were estimated from this distribution. Differences across session blocks were assessed using the same permutation procedure applied pairwise across blocks. Resulting *p*-values were corrected for multiple comparisons using Bonferroni correction. Comparisons to reference stochastic models were performed using matched-N Monte Carlo resampling procedures, as described above.

##### Reward rate - Transition entropy correlation

Associations between reward rate and global transition entropy were quantified across session blocks using Spearman’s rank correlation on block-level averages. Statistical significance was assessed using permutation tests (5,000 permutations) in which the pairing between entropy and reward-rate values was randomly permuted across blocks. Empirical correlation coefficients were compared to the resulting permutation-based null distribution to obtain two-sided *p*-values.

##### Local transition entropy

Local entropy was computed for each source dispenser. For a given location *i*, outgoing transition counts were normalized to obtain an empirical conditional distribution *p*(*j*∣*i*) over subsequent locations. Local transition entropy was defined as:

$$H_{\text{local}}\left(i\right)=-\sum_{j}p\left(j|i\right)\log_{2}\left(p\left(j|i\right)\right)\text{.}$$

Local entropy was computed separately within each session block using pooled transition counts restricted to the standardized eight-choice window. Locations with fewer than 10 outgoing transitions within a block were excluded from statistical analyses but retained in descriptive visualizations where appropriate. This threshold was chosen to ensure sufficient data for reliable entropy estimation across the nine possible transition targets. To assess whether local entropy profiles differed across dispenser locations beyond what would be expected by relabeling, permutation tests (5,000 permutations) were performed by randomly shuffling location labels within each session block while preserving the set of entropy values and the pattern of missing data. Two complementary statistics were evaluated: (i) the variance across locations of mean local entropy averaged across session blocks, and (ii) the variance across locations of linear entropy slopes across session blocks. Empirical *p*-values were estimated from the permutation-based null distributions.

##### Transition matrix analyses

Transition probabilities between dispenser states were estimated from observed transitions within the standardized eight-choice window. For each dispenser *i*, the probability of transitioning to dispenser *j* was defined as:

$$P\left(i\to j\right)=\frac{C_{ij}}{\sum_{k}C_{ik}}\text{,}$$

where *C*_*ij*_ denotes the number of observed transitions from *i* to *j*. For each behavioral partition (clusters and session blocks), directed transition matrices were constructed from pooled transitions and row-normalized to represent empirical transition probabilities.

Pairwise similarity between transition matrices was quantified using Pearson correlation computed over vectorized matrix entries. By default, correlations were computed over off-diagonal entries only, such that self-transitions did not contribute to similarity estimates. Statistical significance was assessed using row-wise permutation tests (5,000 permutations): at each iteration, one of the two matrices was randomly selected and its off-diagonal entries were permuted within each row, preserving the set of outgoing transition probabilities for each dispenser. Empirical correlations were compared to the resulting permutation-based null distributions to obtain two-sided permutation *p*-values, which were Bonferroni-corrected across all unique matrix pairs. In parallel, matrix dissimilarity was quantified using Euclidean distance computed over the same set of compared entries. To facilitate comparisons across partitions, Euclidean distances were additionally normalized by the square root of the number of compared entries.

##### Transition-level non-reward probability

To characterize cluster-specific differences at the level of individual spatial transitions, we quantified a transition-level non-reward probability within the standardized eight-choice window. For each cluster, all directed transitions were extracted across trials, and for each transition we computed the proportion of non-rewarded occurrences (defined as revisits or home returns) relative to the total number of occurrences of that transition. Analyses were restricted to transitions shared by Clusters 0 and 1 to ensure direct comparability. Similarity in transition-level non-reward probability profiles between clusters was assessed using Spearman’s rank correlation.

To account for differences in transition usage frequency, a frequency-weighted mean non-reward probability was additionally computed for each cluster as the total number of non-rewarded occurrences divided by the total number of occurrences across shared transitions.

##### Sequence diversity

Sequence diversity was quantified using the normalized Shannon diversity index, computed from the relative frequencies of unique sequences:

$$H_{\text{norm}}=\frac{-\sum_{i=1}^{N}p_{i}\;\log_{2}p_{i}}{\log_{2}\;N}$$

where N is the total number of unique sequences, and *p*_*i*_ denotes the relative frequency of sequence *i*, defined as the number of occurrences of that sequence divided by the total number of sequences. This normalization constrains diversity values between 0, corresponding to exclusive reliance on a single sequence, and 1, corresponding to uniform use of all observed sequences.

Sequence diversity was computed separately for each session block and restricted to trials without non-rewarded events, as defined above.

##### Sequence similarity

To assess structural similarity between full choice sequences, we quantified the overlap in transition structure across sequences. For each sequence, the set of directed transitions was extracted, and pairwise similarity between sequences was computed using the Jaccard similarity index:

$$J\left(A,B\right)=\frac{\left|A\cap B\right|}{\left|A\cup B\right|}$$

where A and B denote the transition sets of two sequences. The Jaccard index ranges from 0, indicating no shared transitions, to 1, indicating complete overlap.

Analyses were restricted to trials without non-rewarded events. Jaccard similarity was first computed globally across all trials without non-rewarded events to characterize overall sequence overlap. Similarity was then computed within each session block by restricting comparisons to sequences belonging to the same block. For statistical analyses, each sequence was assigned a single summary value equal to its mean similarity to all other sequences within the same block, and these values were compared across blocks using Kruskal-Wallis tests followed by Dunn’s post hoc comparisons with Bonferroni correction. To assess similarity between session blocks, each block was additionally represented by the set of unique transitions expressed across its sequences, and pairwise Jaccard similarity was computed between these block-level transition sets.

##### Dominant sequence frequency

The dominant sequence was identified as the most frequently occurring sequence across all trials without non-rewarded events. For each session block, its frequency of use was computed as the proportion of trials in which the dominant sequence was executed.

##### New sequence ratio

The New Sequence Ratio (NSR) was defined as the number of unique sequences observed in a given session block that had not been encountered in any previous block, expressed as a fraction of all unique sequences observed in that block. This metric was calculated iteratively by maintaining a cumulative set of previously observed sequences across blocks. Analyses were restricted to trials without non-rewarded events.

##### Reused sequence ratio

The Reused Sequence Ratio (RSR) was defined as the proportion of sequences in each session block that had already been observed in earlier blocks, restricted to trials without non-rewarded events. Reused sequences were identified by comparing sequences in the current block to the cumulative set of previously encountered sequences, and the ratio was defined as the number of reused sequences divided by the total number of sequences in that block.

#### Structure-only agent and reward analyses

##### Structure-only agent

To assess how much of the observed behavior could be accounted for by transition structure alone, empirical behavior was compared to a structure-only agent implemented as a first-order Markov model. Analyses were performed separately for each animal. For each animal and empirical partition of interest, directed transition counts were computed from empirically observed dispenser-to-dispenser transitions within the standardized eight-choice window and row-normalized to obtain a transition probability matrix over the nine dispenser states (home dispenser plus eight peripheral dispensers).

Simulated trials always began at the home dispenser and consisted of eight successive post-home choices. At each step, the next dispenser was sampled probabilistically from the row of the transition matrix associated with the current location, such that

$$P\left(X_{t+1}=j|X_{t}=i\right)=T_{ij}\text{.}$$

Thus, the agent had access to the current dispenser and its empirically observed outgoing transition probabilities, but not to the visit history of the ongoing trial, i.e., which dispensers had already been visited earlier in that trial. In this way, within-trial visit history was excluded by restricting the agent’s information to the current state alone. Because choices were generated solely from the current location and its outgoing transition probabilities, the model served as a structure-only baseline.

For each animal and session block, a pool of 5,000 simulated Markov trials was generated. Comparisons with empirical data were then performed using the same matched-N resampling logic described above for stochastic reference models, with resampling from the corresponding block-specific structure-only pool. For analyses pooling all trials across sessions, simulated datasets were assembled by drawing, for each session block, the same number of trials as in the corresponding empirical block, and concatenating across blocks.

##### Reward probability across trial progression

To characterize how choice outcomes evolved within the ongoing trial, we quantified the probability of selecting a rewarded dispenser as a function of trial progression. Analyses were based on the standardized eight-choice window following the initial home visit and included all trials. Choice-level analyses therefore included up to eight observations per trial within the standardized post-home window. For each choice within this window, trial progression was defined as the number of distinct peripheral dispensers already visited earlier in the same trial, denoted *k*. Thus, *k = 0* for the first post-home choice and increased by one each time a new peripheral dispenser was sampled.

A choice was scored as rewarded if it targeted a peripheral dispenser that had not yet been visited within the current trial, and as non-rewarded otherwise, including revisits to previously visited peripheral dispensers and returns to the home dispenser. For a given empirical partition, *P(reward | k)* was computed as the proportion of rewarded choices among all choices made at a given value of *k*. Curves were first computed on the full set of empirical trials pooled across sessions and then recomputed separately within each of the six session blocks defined above. Because the standardized analysis window comprised eight post-home choices, curves were evaluated over *k* = 0-7.

Uncertainty on empirical *P(reward | k)* curves was estimated by non-parametric bootstrap resampling at the trial level (1,000 resamples with replacement). Structure-only curves were estimated from matched-N resamples of the corresponding simulation pools. To describe the difference between empirical and structure-only behavior, Δ*P(reward | k)* was computed as the pointwise difference between empirical and simulated curves.

##### Logistic regression analysis

To quantify how reward outcome varied with trial progression, experience, and agent identity, logistic regression models were fit separately for each animal. Each observation corresponded to a single choice within the standardized eight-choice window, and the dependent variable was binary reward outcome (1 = rewarded choice; 0 = non-rewarded choice).

Predictors included trial progression (k), session block, and agent identity (empirical versus structure-only; coded as 0 = empirical, 1 = structure-only), along with all two-way and three-way interaction terms (k × block, k × agent, block × agent, and k × block × agent). For these analyses, choice-level rows were constructed both from empirical trials and from matched-N structure-only simulated trials sampled within each session block. Session block was treated as an ordered variable spanning the six consecutive five-session blocks, and both trial progression and session block were mean-centered prior to model fitting. Model fitting was inspected for convergence, and the distribution of observations across agents and session blocks was examined to ensure adequate data support.

##### Reward rate vs. structure-only agent

To determine how much of the observed reward rate could be accounted for by transition structure alone, empirical reward rates were compared with those generated by the same structure-only agent. Reward rate was defined as the proportion of rewarded choices within the standardized eight-choice window, i.e. the fraction of choices directed to previously unvisited peripheral dispensers.

For each animal and session block, empirical reward rate was estimated from all empirical trials in the corresponding block. Uncertainty on empirical reward rate was estimated using non-parametric bootstrap resampling at the trial level (1,000 resamples with replacement). Structure-only reward rates were estimated from matched-N resamples of the corresponding block-specific simulation pools.

To summarize how much of empirical reward rate was captured by the structure-only agent, we computed the ratio RR_agent / RR_empirical. To quantify the residual component not explained by transition structure alone, we additionally computed ΔRR = RR_empirical – RR_agent, expressed in percentage points. These comparisons were used descriptively to assess the extent to which transition structure accounted for observed reward rate and the magnitude of the remaining contribution beyond transition structure.

#### Markov-model analyses

All Markov analyses were restricted to trials without non-rewarded events (no revisits, no omissions, and no home returns before completion), as defined above, and were performed separately for each animal. Analyses were first conducted on the full set of eligible trials pooled across sessions and then repeated independently within each session block to characterize experience-dependent changes. Each trial was represented as a fixed-length sequence of states comprising the home dispenser (state 0) followed by the eight peripheral dispensers (states 1-8).

##### Transition matrix estimation

For a given set of training sequences, directed transition counts *C*_*ij*_ were computed for all observed transitions from state *i* to state *j*. Counts were row-normalized to obtain a first-order transition probability matrix *T*:

$$T_{ij}=\frac{C_{ij}}{\sum_{k}C_{ik}}\text{.}$$

Rows with zero outgoing counts were left as zero vectors. No smoothing was applied. Given a current state *i*, the model’s point prediction for the next state was defined as

$$\hat{j}=\arg\underset{j}{\max}T_{ij}\text{.}$$

##### Local predictability

Local predictability was quantified as transition-level accuracy using trial-level K-fold cross-validation (KFold; shuffled splits; fixed random seed). The default number of folds was K = 10. When the number of available sequences N in a given analysis subset was smaller than 10, the number of folds was adjusted to K = min(10,N).

For each fold, the transition matrix *T* was estimated exclusively from the training sequences and evaluated on held-out sequences. Transition-level accuracy was computed across all successive state pairs in the test sequences by calculating the proportion of transitions for which the predicted next state matched the observed next state. Fold-wise accuracies were averaged to obtain cross-validated performance estimates.

Confusion matrices were additionally computed within each fold to assess state-specific prediction performance and averaged across folds for visualization, with the home state excluded.

##### Sequence-level predictability

Sequence-level predictability was quantified as full-sequence reconstruction accuracy and was evaluated using the same cross-validation procedure described above.

Within each fold, the transition matrix *T* was estimated from the training sequences. For each held-out trial, a predicted sequence was generated using deterministic greedy decoding: starting from the observed initial state (home), the model iteratively selected the maximum-probability transition (*arg*⁡*max*⁡_*j*_*T*_*ij*_) at each step over the fixed eight-choice window.

Because all analyzed trials had identical length (home plus eight peripheral choices), predicted and observed sequences had equal length. A sequence was scored as correctly predicted only if the reconstructed sequence exactly matched the observed sequence across the full standardized window. Sequence-level accuracy was defined as the proportion of correctly reconstructed sequences in the held-out data and was averaged across folds.

##### Comparison to stochastic reference models

To determine whether Markov predictability exceeded stochastic expectations, empirical cross-validated performance was compared with null distributions derived from the uniform random-choice and spatially constrained random-walk models described above (variants disallowing non-rewarded events).

For each null simulation, sequences were resampled with replacement to match the empirical sample size (matched-N), and Markov performance was evaluated using the same cross-validation procedure (matched-K) as applied to the empirical data. Repeating this procedure across simulations yielded null distributions of cross-validated mean accuracy.

Statistical significance was assessed using one-sided Monte Carlo tests (empirical performance > null expectation), as described above. *p*-values were Bonferroni-corrected across the two null models.

##### Session-block analyses and statistics

To characterize experience-dependent changes in Markov predictability, the full analysis pipeline (transition matrix estimation, cross-validation, and null comparisons) was repeated independently within each session block using only eligible trials belonging to that block.

Differences in local and sequence-level predictability across session blocks were summarized using Kruskal-Wallis tests applied to fold-level cross-validated accuracies, which are reported here as summary measures of predictive performance. When significant, pairwise comparisons were performed using Dunn’s post hoc tests with Bonferroni correction for multiple comparisons.

#### Successor representation analyses

All successor representation (SR) analyses were restricted to trials without non-rewarded events (no revisits, no omissions, and no home returns before completion), as defined above, and were performed separately for each animal. Analyses were first conducted on the full set of eligible trials pooled across sessions and then repeated independently within each session block to characterize experience-dependent changes. Each trial was represented as a fixed-length state sequence comprising the home dispenser (state 0) followed by the eight peripheral dispensers (states 1-8).

##### Successor representation construction

For each analysis subset, the first-order transition probability matrix *T* was estimated from training sequences as described in the Markov-model analyses. The successor representation matrix was then computed as

$$M={\left(I-\gamma T\right)}^{-1}\text{,}$$

where *γ*∈(0,1) is the discount factor.

##### Multi-step predictive accuracy

To quantify state-dependent multi-step predictability, we evaluated whether the successor representation (SR) assigns higher scores to dispensers that will be visited within the next H steps than to dispensers that will not. Predictive accuracy was assessed using receiver operating characteristic area under the curve (ROC AUC) across horizons H = 1-5. Horizons were restricted to H = 1-5 to ensure discriminative evaluation, as the number of yet-unvisited dispensers decreases monotonically within trials without non-rewarded events, reducing the number of competing candidates at larger horizons.

For each held-out sequence and time step *t*, the current state *i*=*x*_*t*_ defined the set of visited locations *V*_*t*_={*x*_0_,…,*x*_*t*_}. Candidate targets were restricted to yet-unvisited peripheral dispensers,

$$C_{t}=\left\{c\in \left\{1,\ldots ,8\right\}|\;c\notin V_{t}\right\}\text{,}$$

explicitly excluding the home state. For a given horizon *H*, the positive set was defined as the dispensers visited within the next H steps,

$$F_{t,H}=\left\{x_{t+1},\ldots ,x_{t+H}\right\}\text{,}$$

Each candidate *c* ∈ *C*_*t*_ was labeled 1 if *c* ∈ *F*_*t*,*H*_ and 0 otherwise. Candidates were scored using the corresponding SR value *M*_*ic*_, reflecting the expected discounted future occupancy of state *c* when starting from *i*. Step-level ROC AUC values were computed by comparing SR scores against binary labels over candidates *C*_*t*_. AUC therefore quantified the probability that a dispenser visited within the next H steps received a higher SR score than a dispenser not visited within that horizon. Steps with fewer than two candidate targets were excluded.

AUC values were aggregated across valid steps within each test fold, and fold-wise means were averaged to obtain cross-validated performance for each horizon. For summary statistics and statistical comparisons, AUC values were additionally averaged across horizons. Global analyses (pooled across sessions) used 10-fold cross-validation. Within-session-block analyses used K = min(10,N) cross-validation folds to accommodate smaller sample sizes.

##### Discount factor selection

The discount factor *γ* was selected based on cross-validated multi-step predictive accuracy computed on eligible trials pooled across sessions. Candidate values *γ* ∈ {0.1,0.2,…,0.9} were evaluated. For each *γ*, mean ROC AUC was computed across horizons H = 1-5 and averaged to obtain a single selection score. The value maximizing this score was identified for each animal. To ensure robustness, we defined a stability range including all *γ* values within 0.01 AUC units of the maximum. The value *γ*=0.7 lay within the stable range for both animals and was therefore used in all subsequent SR analyses (Figure S7A).

##### Low-dimensional SR embedding

To characterize the structure of multi-step predictive profiles across locations, SR matrices were embedded into two dimensions using classical multidimensional scaling (MDS). Pairwise Euclidean distances were computed between rows of the SR matrix (i.e., future-occupancy profiles *M*_*i*·_), yielding a state-by-state distance matrix. Classical MDS was performed via eigen-decomposition of the double-centered squared distance matrix, producing a two-dimensional embedding in which inter-node distances approximate dissimilarity of multi-step predictive profiles.

##### Comparison to stochastic reference models

To assess whether SR-based predictability exceeded stochastic expectations, empirical cross-validated AUC values were compared with null distributions derived from the uniform random-choice and spatially constrained random-walk reference models described above (variants disallowing non-rewarded events).

For each null simulation, sequences were resampled with replacement to match the empirical number of trials (matched-N). Multi-step predictive accuracy was then evaluated using the identical cross-validation procedure as applied to the empirical data. For each simulation replicate, AUC values were averaged across horizons H = 1-5 to yield a single summary statistic.

Statistical significance was assessed using one-sided Monte Carlo tests (empirical performance > null expectation), as described above. *p*-values were Bonferroni-corrected across the two null models.

##### Experience-dependent changes and convergence

To assess experience-dependent changes in SR predictability, the full analysis pipeline was repeated independently within each session block using only eligible trials belonging to that block.

Within each session block, cross-validated AUC values were averaged across horizons H = 1-5 to obtain a single multi-step predictability measure per fold. Differences across session blocks were summarized using Kruskal-Wallis tests applied to fold-level values, reported here as summary measures of predictive performance. When significant, pairwise comparisons were performed using Dunn’s post hoc tests with Bonferroni correction for multiple comparisons.

To quantify convergence of multi-step predictive structure with experience, SR matrices computed within each session block were compared to the global SR computed from all eligible trials pooled across all thirty sessions, using a normalized Frobenius distance:

$$d\left(M_{\text{block}},M_{\text{global}}\right)=\frac{∥M_{\text{block}}-M_{\text{global}}{∥}_{F}}{∥M_{\text{global}}{∥}_{F}}\text{.}$$

This metric quantifies the magnitude of deviation of each block-specific predictive structure from the overall multi-step organization expressed across sessions.
