# Supplementary material for: Experience-dependent emergence of multi-scale spatial organization during foraging in freely moving marmosets
Source: iScience. 2026 Aug 26;29(9):117294. doi: 10.1016/j.isci.2026.117294 (PMC13544335; doi:10.1016/j.isci.2026.117294)
Supplement: Supplementary file 1 [file mmc1.pdf]

iScience, Volume *29*

Supplemental information

Experience-dependent emergence of multi-scale spatial organization during foraging in freely moving marmosets

Nada El Mahmoudi, Francesca Lanzarini, Farzad Ziaie Nezhad, Deepak Surendran, and Jean Laurens

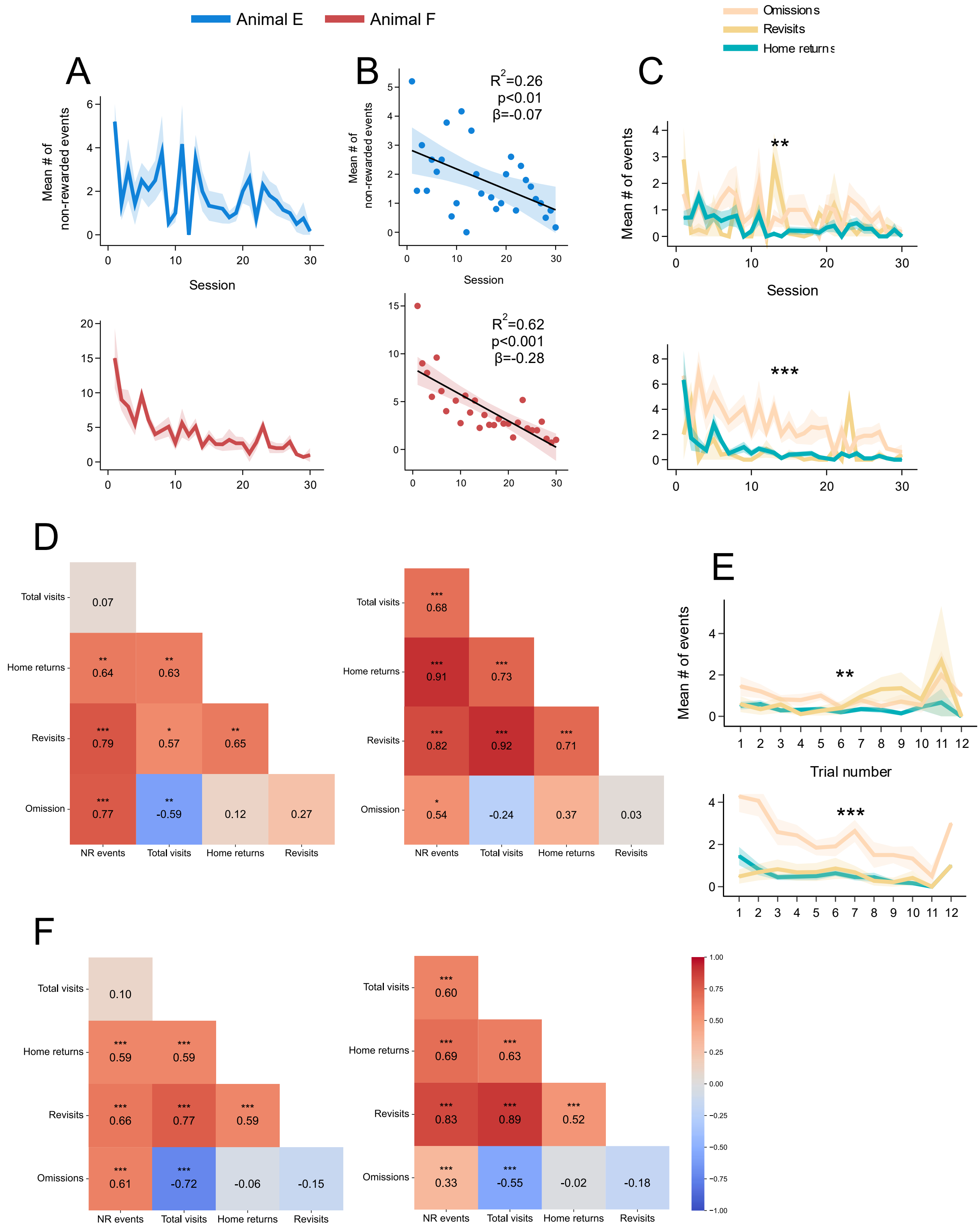

Animal E
Animal F
Omissions
Revisits
Home returns
A
B
C
D
E
F
Mean # of non-rewarded events
Session
Mean # of events
R²=0.26
p<0.01
β=-0.07
R²=0.62
p<0.001
β=-0.28
**
***
Total visits
Home returns
Revisits
Omission
Omissions
NR events
Trial number
0.07
0.64
0.63
0.79
0.57
0.65
0.77
-0.59
0.12
0.27
0.68
0.91
0.73
0.82
0.92
0.71
0.54
-0.24
0.37
0.03
0.10
0.59
0.59
0.66
0.77
0.59
0.61
-0.72
-0.06
-0.15
0.60
0.69
0.63
0.83
0.89
0.52
0.33
-0.55
-0.02
-0.18

Figure S1. Extended analyses of non-rewarded events.
Related to Figure 1.

A. Session-wise evolution of non-rewarded events.
Line plots showing the mean number of non-rewarded events per session for Animal E (*top*) and Animal F (*bottom*). Shaded areas indicate the standard error of the mean (SEM) across trials within each session.

B. Linear regression of non-rewarded events across sessions.
Scatter plots showing the relationship between session number and the mean number of non-rewarded events for Animal E (*top*) and Animal F (*bottom*). Each point corresponds to a session mean. Solid lines indicate best-fit linear regressions, and shaded areas represent 95% confidence intervals. Reported statistics (R^2, p-values, and slopes β) correspond to linear regression analyses showing a significant decrease in non-rewarded events across sessions in both animals.

C. Session-wise evolution of revisits, omissions, and home returns.
Session-wise mean counts of revisits, omissions, and home returns across sessions for Animal E (*top*) and Animal F (bottom). Colored lines correspond to the three event types. Kruskal-Wallis tests applied to session-wise mean counts revealed significant differences across event types in both animals (Animal E: $H = 13.06$, $p < 0.01$; Animal F: $H = 39.05$, $p < 0.001$), indicating distinct and non-uniform trajectories of different non-rewarded events over sessions.

D. Session-level covariation between non-rewarded event types.
Pearson correlation matrices computed on session-averaged metrics, including non-rewarded events (NR events), revisits, omissions, home returns, and total number of dispenser visits, for Animal E (*left*) and Animal F (*right*). Cell values report Pearson correlation coefficients (r), with color indicating correlation strength and sign. Statistical significance was assessed using Bonferroni-corrected p-values for multiple comparisons. Revisits and home returns were strongly positively correlated with each other and with total visits, whereas omissions showed weaker or inconsistent associations.

E. Trial-wise evolution of revisits, omissions, and home returns.
Trial-wise mean counts of revisits, omissions, and home returns as a function of trial position within sessions for Animal E (*top*) and Animal F (*bottom*). Kruskal-Wallis tests revealed significant differences across event types at the trial level in both animals (Animal E: $H = 11.41$, $p < 0.01$; Animal F: $H = 15.82$, $p < 0.001$), indicating distinct within-session dynamics of different non-rewarded events.

F. Trial-level covariation between non-rewarded event types.
Pearson correlation matrices computed on trial-averaged metrics for Animal E (*left*) and Animal F (*right*), including non-rewarded events (NR events), revisits, omissions, home returns, and total dispenser visits. Statistical significance was assessed as in panel D. Trial-level covariation mirrored session-level results, with strong positive associations between revisits, home returns, and total visits, and weaker or negative associations involving omissions.

Significance levels: $^*p < 0.05$; $^{**}p < 0.01$; $^{***}p < 0.001$.

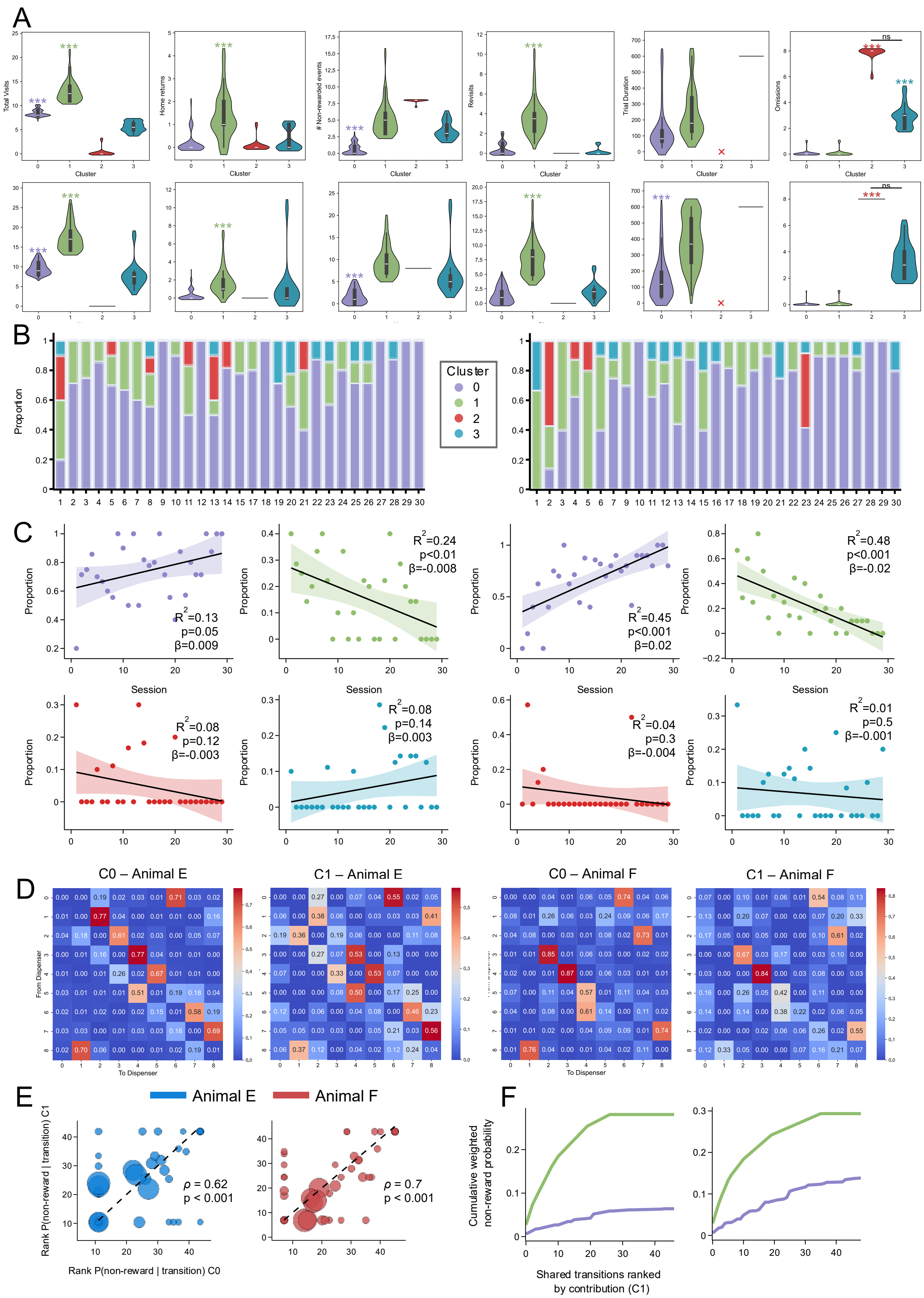
A
Total Visits
Home returns
Non-rewarded events
Revisits
Trial Duration
Omissions
Cluster
ns
B
Proportion
Cluster
0
1
2
3
C
Proportion
Session
R^2=0.13
p=0.05
β=0.009
R^2=0.24
p<0.01
β=-0.008
R^2=0.45
p<0.001
β=0.02
R^2=0.48
p<0.001
β=-0.02
R^2=0.08
p=0.12
β=-0.003
R^2=0.08
p=0.14
β=0.003
R^2=0.04
p=0.3
β=-0.004
R^2=0.01
p=0.5
β=-0.001
D
C0 – Animal E
C1 – Animal E
C0 – Animal F
C1 – Animal F
From Dispenser
To Dispenser
E
Animal E
Animal F
Rank P(non-reward | transition) C1
Rank P(non-reward | transition) C0
ρ = 0.62
p < 0.001
ρ = 0.7
p < 0.001
F
Cumulative weighted
non-reward probability
Shared transitions ranked
by contribution (C1)

Figure S2. Clustering analyses reveal distinct trial execution profiles and shared transition structure.
Related to Figure 2.

A. Behavioral differences across clusters.
Violin plots showing the distribution of key trial-level behavioral variables across the four clusters (0-3) identified by unsupervised k-means clustering, for Animal E (*top row*) and Animal F (*bottom row*). Variables include total number of dispenser visits, number of omissions, number of revisits, number of home returns, and trial duration. Horizontal lines indicate medians and the violin width reflects the density of observations. Statistical differences across clusters were assessed using Kruskal-Wallis tests followed, when significant, by Dunn post hoc comparisons with Bonferroni correction. Asterisks indicate significant differences between clusters (*$p < 0.05$; **$p < 0.01$; ***$p < 0.001$). Red crosses denote clusters for which no data were available.

B. Evolution of cluster proportions across sessions.
Stacked bar plots showing the proportion of trials assigned to each cluster as a function of session number for Animal E (*left*) and Animal F (*right*). Each bar corresponds to one session, with colors indicating cluster identity.

C. Linear regression of cluster proportions across sessions.
Scatter plots showing session-wise proportions of trials belonging to each cluster as a function of session number, with linear regression fits overlaid for Animal E (*left panels*) and Animal F (*right panels*). Shaded areas indicate 95% confidence intervals. Reported statistics correspond to linear regression analyses (R^2, p-values, and slopes β). The proportion of Cluster 0 trials increased across sessions, whereas the proportion of Cluster 1 trials decreased. In contrast Clusters 2 and 3 showed no systematic change over time.

D. Dispenser-to-dispenser transition matrices.
Dispenser-to-dispenser transition probability matrices for Cluster 0 (C0) and Cluster 1 (C1) shown for Animal E (*left panels*) and Animal F (*right panels*). Each matrix summarizes transition probabilities aggregated across all trials assigned to the corresponding cluster. Rows correspond to the current dispenser and columns to the next dispenser. Color intensity represents transition probability. Transition matrices were highly similar across clusters in both animals, as quantified by strong Pearson correlations (Animal E: $r = 0.88$; Animal F: $r = 0.91$; permutation tests, Bonferroni-corrected, $p < 0.001$), indicating reliance on largely overlapping transition sets. Despite this structural similarity, Euclidean distances between matrices (Animal E: 0.90; Animal F: 0.84) revealed quantitative differences in transition strength, reflecting differential weighting of shared transitions across clusters.

E. Rank-rank correspondence of transition-specific non-rewarded probabilities across clusters.
Rank-rank plots comparing transition-specific non-rewarded probabilities between Cluster 0 and Cluster 1 for Animal E (left) and Animal F (right). For each transition expressed in both clusters (Animal E: 47 transitions; Animal F: 49 transitions), non-rewarded probability was defined as the proportion of occurrences of that transition resulting in a revisit or a home return. Each point corresponds to one transition, with marker size proportional to its overall frequency. Ranks were computed independently within each cluster. The dashed line ($x = y$) indicates perfect correspondence in ranking. Spearman correlation coefficients (ρ) and p-values are reported and indicate that transitions prone to non-rewarded outcomes in one cluster tended to occupy similar ranks in the other, reflecting that such transitions were largely shared between clusters.

F. Cumulative contribution of shared transitions to non-rewarded outcomes.
Cumulative curves showing how shared transitions contribute to the overall non-rewarded probability in Cluster 0 and Cluster 1 for Animal E (*left*) and Animal F (*right*). For each transition expressed in both clusters, contribution was computed as the product of its usage frequency and its transition-specific non-rewarded probability. Transitions are ranked by their contribution in Cluster 1. Shared transitions accounted for the vast majority of non-rewarded events in Cluster 1 (Animal E: 96%; Animal F: 94%). The steeper accumulation observed for Cluster 1 reflects heavier reliance on transitions intrinsically more likely to produce non-rewarded outcomes, resulting in substantially higher frequency-weighted non-rewarded probabilities than in Cluster 0 (Animal E: 28.1% vs. 6.4%; Animal F: 29.4% vs. 13.8%). These results indicate that elevated non-rewarded event rates in Cluster 1 arise from differential weighting of a common transition repertoire rather than from the use of cluster-specific transitions.

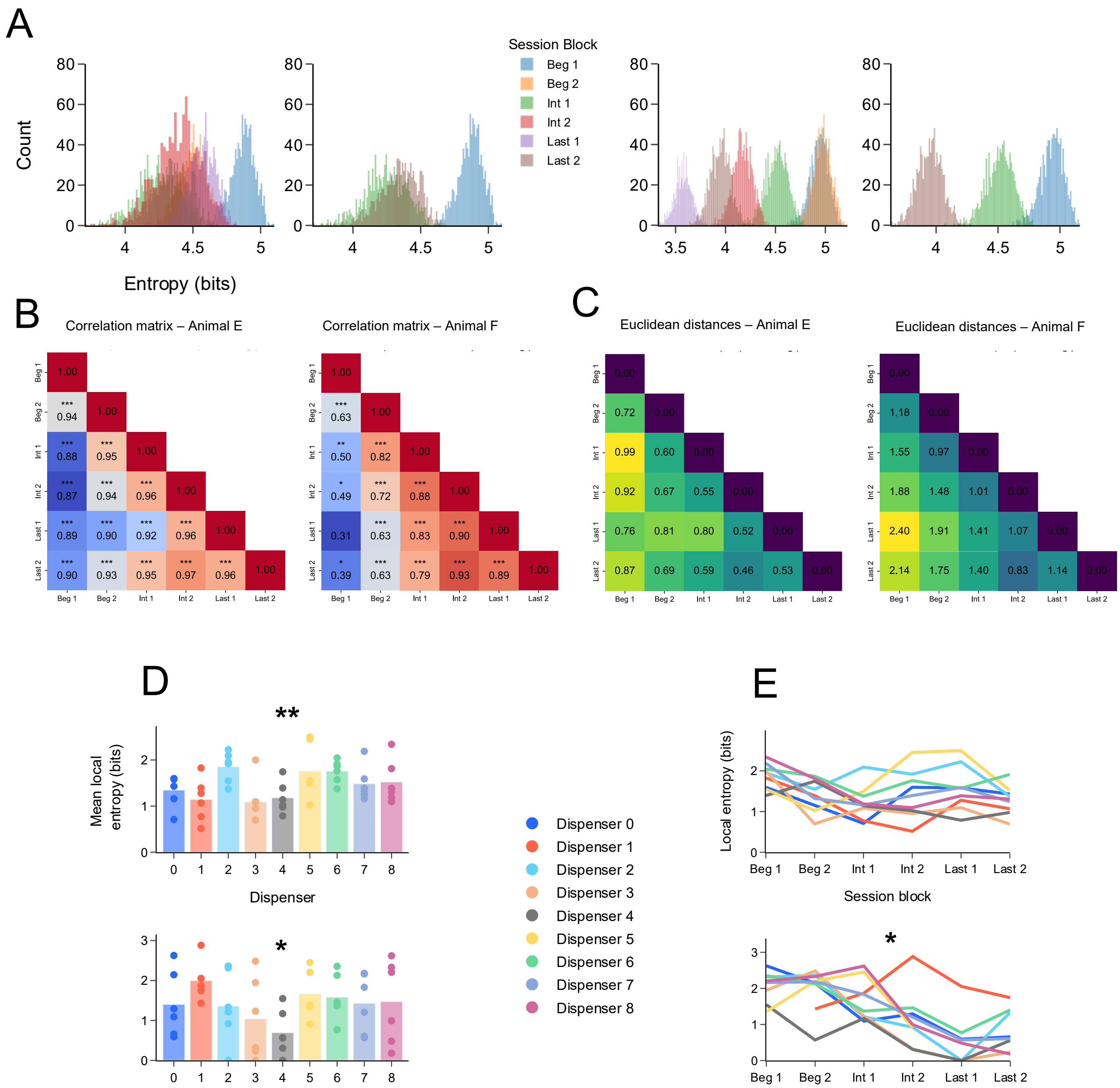
A
Count
Entropy (bits)
Session Block
Beg 1
Beg 2
Int 1
Int 2
Last 1
Last 2
B
Correlation matrix – Animal E
Correlation matrix – Animal F
C
Euclidean distances – Animal E
Euclidean distances – Animal F
Beg 1
Beg 2
Int 1
Int 2
Last 1
Last 2
D
Mean local entropy (bits)
Dispenser
E
Local entropy (bits)
Session block
Dispenser 0
Dispenser 1
Dispenser 2
Dispenser 3
Dispenser 4
Dispenser 5
Dispenser 6
Dispenser 7
Dispenser 8

Figure S3. Extended analyses of experience-dependent changes in the transition repertoire.
Related to Figure 3.
A. Bootstrap distributions of global transition entropy across session blocks.
Bootstrap distributions of global transition entropy computed separately for each session block are shown for Animal E (*left*) and Animal F (*right*). For each animal, the left subpanel displays bootstrap distributions for all session blocks (Beg 1, Beg 2, Int 1, Int 2, Last 1, Last 2), while the right subpanel shows a subset of representative blocks to facilitate visual comparison across stages of experience. Bootstrap distributions were obtained by resampling trial sequences with replacement within each block and recomputing entropy for each resample. The progressive shift toward lower entropy values with experience indicates increasing concentration of transition probabilities.
B. Correlation between block-wise transition matrices.
Pearson correlation matrices between dispenser-to-dispenser transition matrices computed for all pairs of session blocks are shown for Animal E (*left*) and Animal F (*right*). Correlations quantify similarity in the relative structure of transitions across blocks. Cell values report Pearson correlation coefficients (r), with color indicating correlation strength. Statistical significance was assessed using permutation tests with Bonferroni correction for multiple comparisons. Overall, transition matrices were highly correlated across session blocks, indicating preservation of relative transition structure over experience.
C. Euclidean distance between block-wise transition matrices.
Heatmaps showing Euclidean distances between block-specific transition matrices for Animal E (*left*) and Animal F (*right*). Distances quantify differences in absolute transition probabilities across session blocks. Distances were larger between early blocks and decreased across later blocks, indicating progressive stabilization of transition weights over experience.
D. Mean local transition entropy across dispensers.
Bar plots show the mean local transition entropy for each dispenser, averaged across session blocks, for Animal E (*top*) and Animal F (*bottom*). Individual data points correspond to block-wise entropy estimates. Local entropy is defined as the entropy of transitions originating from a given dispenser and captures location-specific transition selectivity. Heterogeneity across dispensers was assessed using a permutation test comparing the variance of mean entropy values across nodes to a null distribution obtained by shuffling dispenser identities within blocks. This analysis revealed significant heterogeneity in node selectivity for both Animal E ($p<0.01$) and Animal F ($p <0.05$).
E. Evolution of local transition entropy across session blocks.
Line plots show the evolution of local transition entropy across session blocks for each dispenser, for Animal E (*top*) and Animal F (*bottom*). Each colored line corresponds to one dispenser. Breaks in trajectories indicate session blocks in which fewer than 10 outgoing transitions were observed for a given dispenser, and local entropy was therefore not estimated. To assess heterogeneity in experience-dependent refinement, linear slopes relating local entropy to session block were computed for each dispenser, and the variance of observed slopes was compared to a null distribution obtained by shuffling dispenser identities within blocks. This analysis revealed no significant heterogeneity in temporal refinement across dispensers for Animal E ($p = 0.15$), but significant heterogeneity for Animal F ($p<0.05$), indicating node-specific differences in experience-related changes in transition selectivity.

Significance levels: *$p < 0.05$; **$p < 0.01$; ***$p < 0.001$.

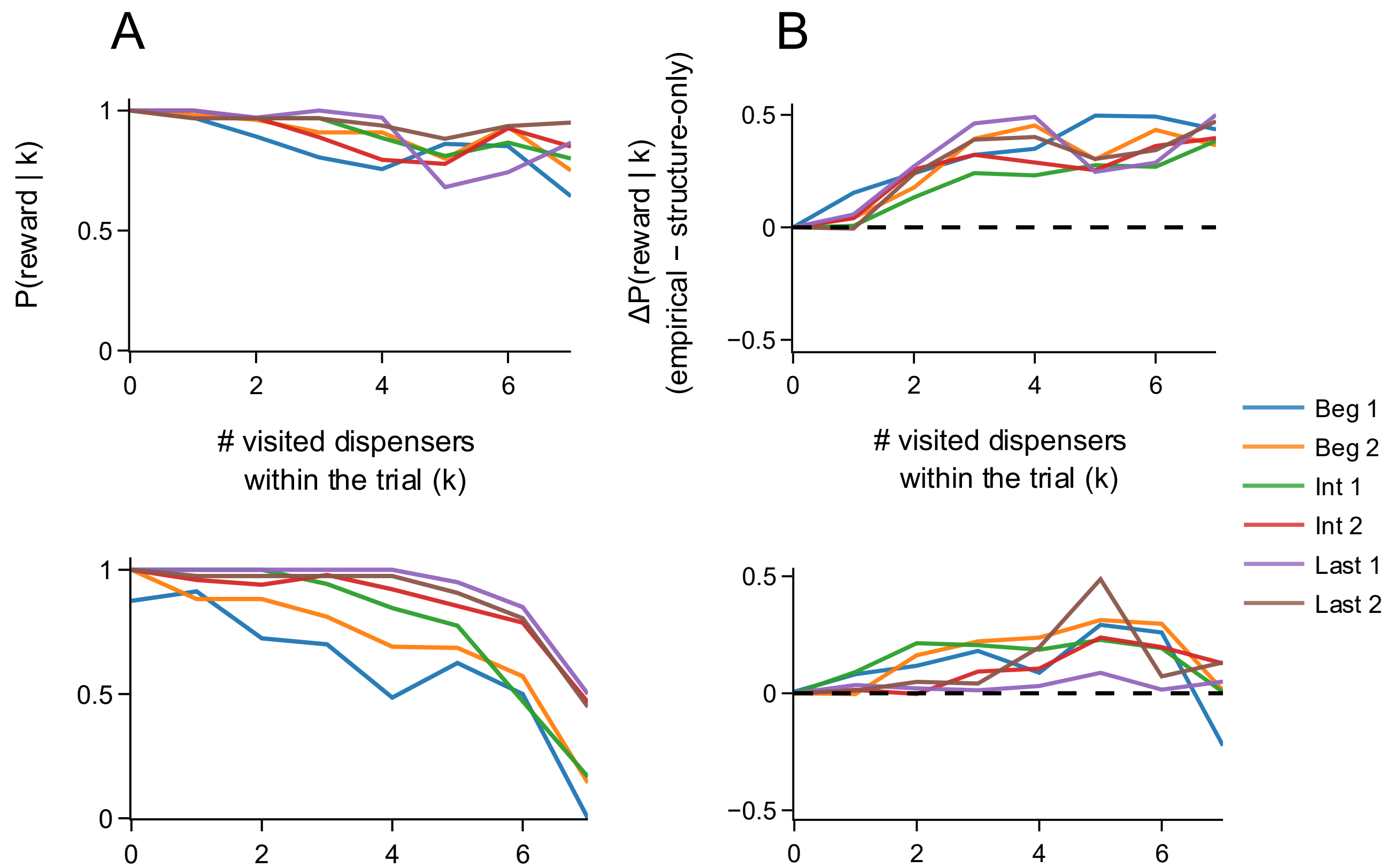

Figure S4. Experience-dependent changes in within-trial reward probability and empirical advantage over the structure-only agent.
Related to Figure 4.

A. Session-block-specific reward probability during trial progression.
Probability of selecting a rewarded dispenser, *P(reward* | *k),* shown separately for each session block as a function of trial progression, quantified by the number of distinct peripheral dispensers already visited within the ongoing trial (*k*). Curves are shown for empirical behavior only, with colors indicating session block, for Animal E (*top*) and Animal F (*bottom*).

B. Session-block-specific empirical advantage over the structure-only agent.
Difference between empirical and structure-only reward probability curves, *ΔP(reward* | *k),* shown separately for each session block. Positive values indicate an empirical advantage over the structure-only agent beyond transition structure alone. Dashed horizontal lines indicate zero difference. Colors indicate session block, for Animal E (*top*) and Animal F (*bottom*).

Full logistic regression results are reported in **Table S3**.

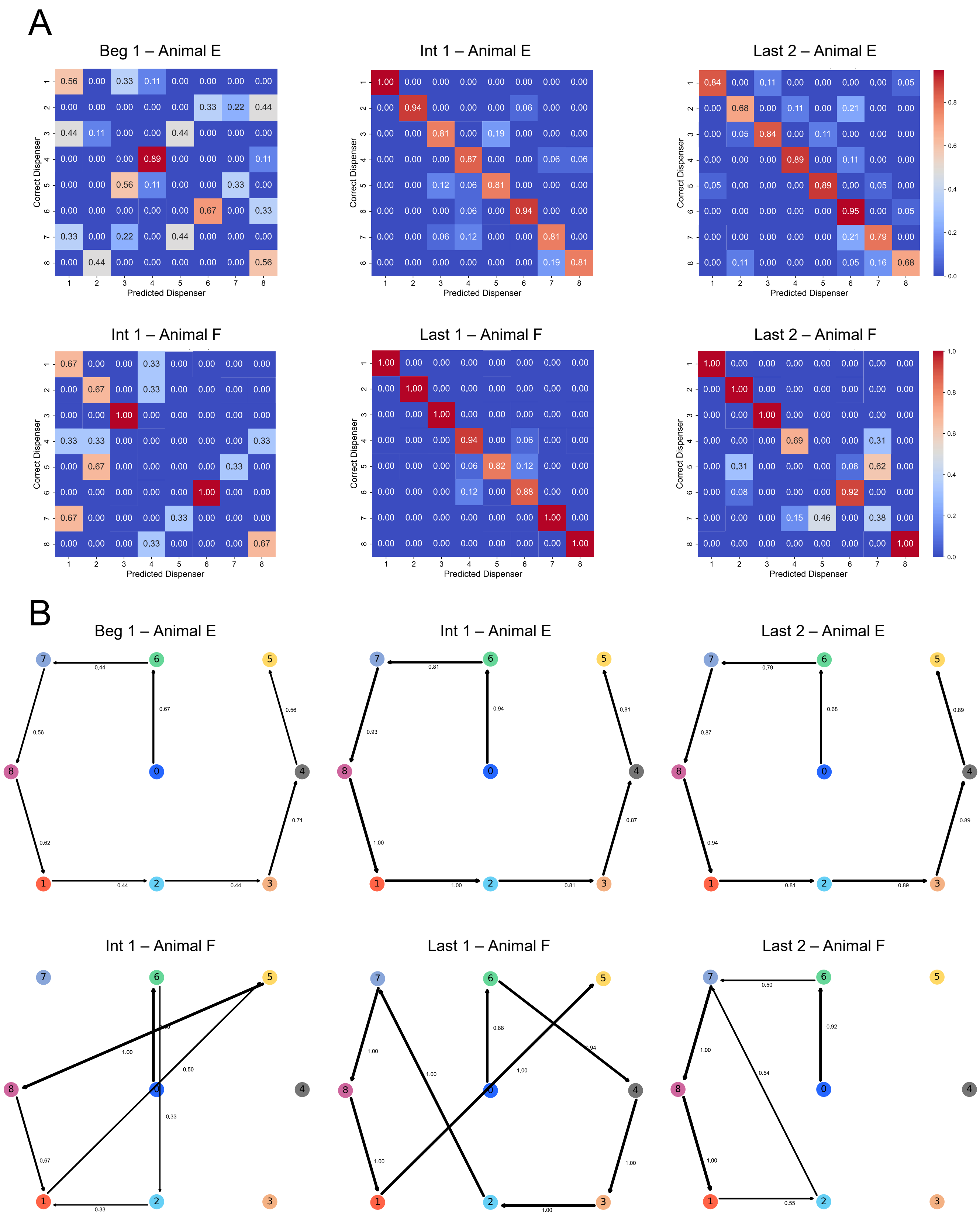
A
Beg 1 – Animal E
Int 1 – Animal E
Last 2 – Animal E
Int 1 – Animal F
Last 1 – Animal F
Last 2 – Animal F
Correct Dispenser
Predicted Dispenser
B
Beg 1 – Animal E
Int 1 – Animal E
Last 2 – Animal E
Int 1 – Animal F
Last 1 – Animal F
Last 2 – Animal F

Figure S5. Experience-dependent changes in local and global Markov predictability. Related to Figure 5.

A. Confusion matrices across session blocks.

Confusion matrices showing predicted versus actual next-dispenser choices for representative session blocks in Animal E (*top row*) and Animal F (*bottom row*). Matrices were obtained from a first-order Markov model trained and evaluated using cross-validation within each session block, restricted to trials without non-rewarded events. Rows correspond to the true next dispenser and columns to the dispenser predicted by the model. Rows are normalized to show the distribution of predictions conditional on the true dispenser. Diagonal entries indicate correct predictions and illustrate the degree of local transition predictability within each block as captured by the Markov model.

B. Markov-predicted visitation sequences across session blocks.

Representative predicted visitation sequences generated by the first-order Markov model for the same session blocks shown in panel A for Animal E (*top row*) and Animal F (*bottom row*). For each block, the model was trained on all eligible trials (without non-rewarded events), and a single predicted sequence was generated by iteratively selecting, at each step, the most probable next transition from the learned transition matrix, starting from the empirically observed first dispenser. Nodes represent dispenser locations arranged according to their spatial positions in the environment. Directed edges indicate the sequence of predicted transitions, with edge thickness proportional to transition probability. Labels indicate transition probabilities.

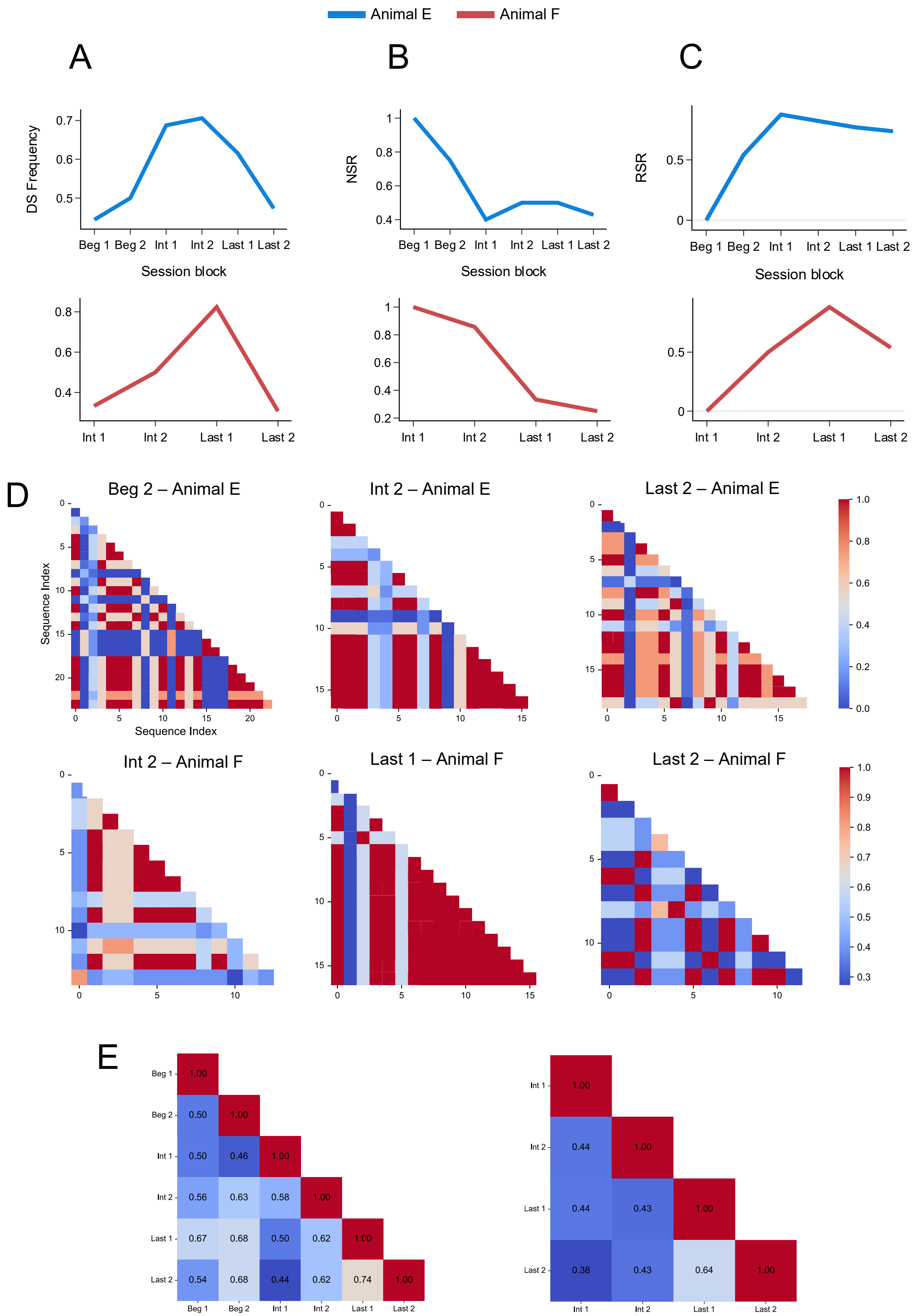
Animal E
Animal F
A
DS Frequency
Session block
Beg 1 Beg 2 Int 1 Int 2 Last 1 Last 2
Int 1 Int 2 Last 1 Last 2
B
NSR
Session block
Beg 1 Beg 2 Int 1 Int 2 Last 1 Last 2
Int 1 Int 2 Last 1 Last 2
C
RSR
Session block
Beg 1 Beg 2 Int 1 Int 2 Last 1 Last 2
Int 1 Int 2 Last 1 Last 2
D
Beg 2 – Animal E
Int 2 – Animal E
Last 2 – Animal E
Sequence Index
Sequence Index
Int 2 – Animal F
Last 1 – Animal F
Last 2 – Animal F
E
Beg 1 1.00
Beg 2 0.50 1.00
Int 1 0.50 0.46 1.00
Int 2 0.56 0.63 0.58 1.00
Last 1 0.67 0.68 0.50 0.62 1.00
Last 2 0.54 0.68 0.44 0.62 0.74 1.00
Beg 1 Beg 2 Int 1 Int 2 Last 1 Last 2
Int 1 1.00
Int 2 0.44 1.00
Last 1 0.44 0.43 1.00
Last 2 0.38 0.43 0.64 1.00
Int 1 Int 2 Last 1 Last 2

Figure S6. Experience-dependent changes in visitation sequence diversity and similarity. Related to Figure 6.

A. Evolution of dominant sequence frequency.

Line plots showing the frequency of the most common complete visitation sequence (DS frequency) within each session block for Animal E (*top*) and Animal F (*bottom*). Values represent the proportion of trials within each block corresponding to the most frequently expressed visitation sequence.

B. New Sequence Ratio across session blocks.

Line plots showing the New Sequence Ratio (NSR) for Animal E (*top*) and Animal F (*bottom*), defined as the proportion of complete visitation sequences within a session block that had not been observed in any preceding block. Higher NSR values indicate that a larger fraction of sequences expressed within a block were newly observed relative to earlier blocks.

C. Reused Sequence Ratio across session blocks.

Line plots showing the Reused Sequence Ratio (RSR) for Animal E (*top*) and Animal F (*bottom*), defined as the proportion of complete visitation sequences within a session block that had been expressed in earlier blocks. Higher RSR values indicate that a larger fraction of sequences expressed within a block corresponded to previously observed visitation sequences.

D. Pairwise similarity between visitation sequences within session blocks.

Heatmaps showing pairwise Jaccard similarity indices between complete visitation sequences within representative session blocks for Animal E (*top row*) and Animal F (*bottom row*). Each cell represents the proportion of shared dispenser-to-dispenser transitions between a pair of sequences (range: 0-1).

E. Pairwise similarity between visitation sequences across session blocks.

Heatmaps showing mean Jaccard similarity between visitation sequences expressed in different session blocks for Animal E (*left*) and Animal F (*right*). Each cell represents the average similarity across all sequence pairs drawn from the corresponding pair of session blocks, illustrating the extent to which transition content is shared across experience.

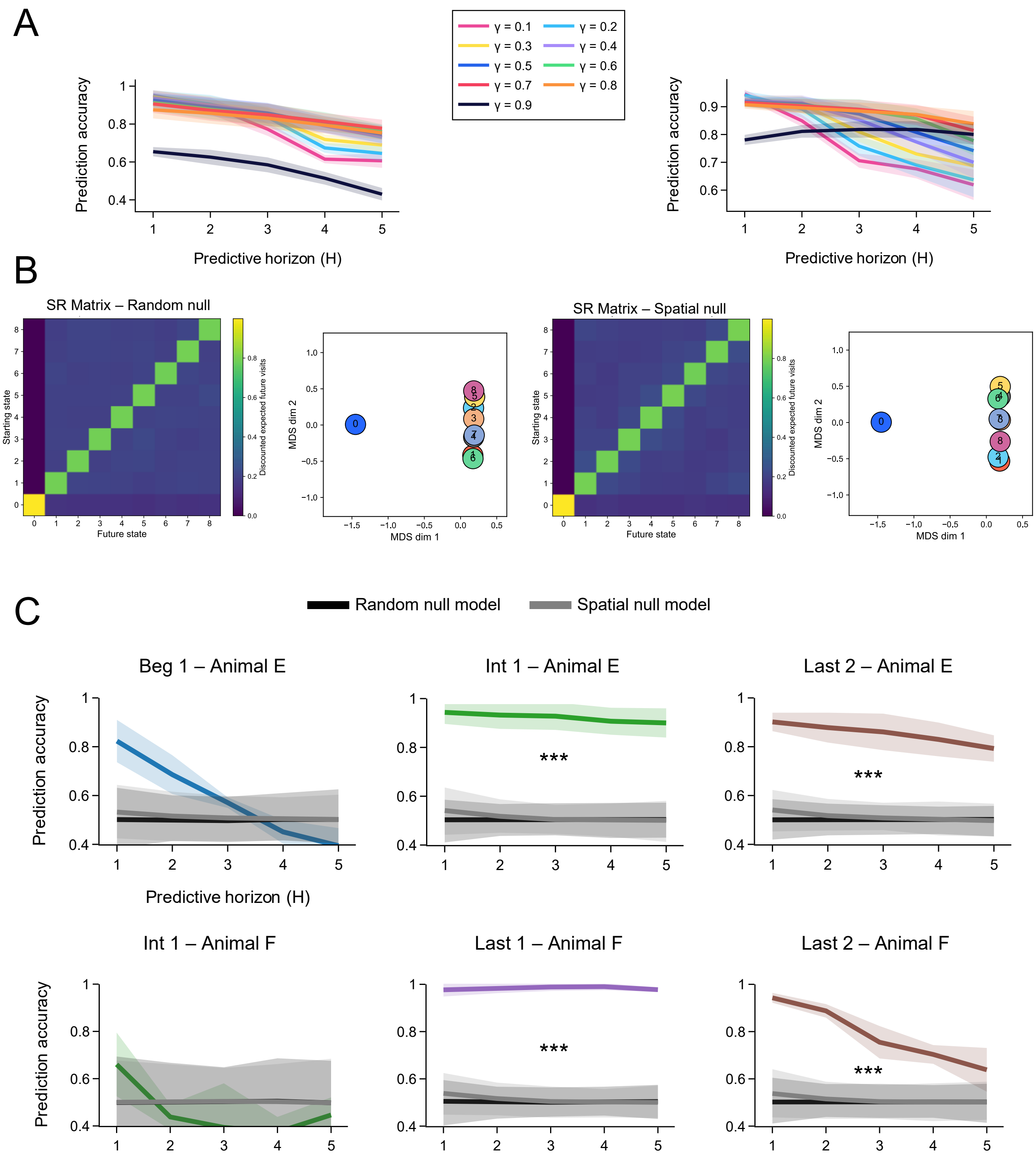

A
γ = 0.1
γ = 0.2
γ = 0.3
γ = 0.4
γ = 0.5
γ = 0.6
γ = 0.7
γ = 0.8
γ = 0.9
Prediction accuracy
Predictive horizon (H)
B
SR Matrix – Random null
SR Matrix – Spatial null
Starting state
Future state
Discounted expected future visits
MDS dim 1
MDS dim 2
C
Random null model
Spatial null model
Beg 1 – Animal E
Int 1 – Animal E
Last 2 – Animal E
Int 1 – Animal F
Last 1 – Animal F
Last 2 – Animal F
***

Figure S7. Extended analyses of the successor representation. Related to Figure 7.

A. Choice of the discount factor (γ).

Line plots showing predictive accuracy of the successor representation (SR) as a function of predictive horizon (H = 1-5) for different values of the discount factor γ (0.1-0.9) for Animal E (*top*) and Animal F (*bottom*). For each γ, a single SR was computed from trials without non-rewarded events and evaluated using cross-validation. Predictive accuracy reflects how well the SR, given the current dispenser, ranked yet-unvisited dispensers according to their likelihood of being visited within the next H steps, and was summarized as area under the ROC curve (AUC). Shaded regions indicate 95% confidence intervals across cross-validation folds. In both animals, predictive performance varied smoothly as a function of γ and exhibited broad regimes of stable performance rather than a sharp optimum. For Animal E, performance remained stable across γ = 0.4-0.7. For Animal F, predictive accuracy increased over a wider range and plateaued across γ = 0.7-0.8. Based on the overlap between these stable regimes and to ensure consistency across animals, γ = 0.7 was used for all successor representation analyses reported in the main text.

B. Successor representation matrices and embeddings for null models.

Successor representation (SR) matrices (*left*) and corresponding low-dimensional embeddings obtained using classical multidimensional scaling (*right*) for two stochastic reference models: a uniform random-choice model (*left pair*) and a spatially constrained random-walk model (*right pair*). In both models, choices were restricted to yet-unvisited dispensers within each trial. Rows correspond to starting dispensers and columns to future dispensers; color indicates discounted expected future visits. Each point in the embedding represents a dispenser location, and distances reflect similarity between SR predictive profiles. In contrast to empirical data, SR embeddings derived from null models exhibited weak or degenerate structure, reflecting the absence of consistent state-dependent multi-step predictive relationships between locations. These embeddings are shown for qualitative comparison and illustrate the geometry expected in the absence of structured predictive organization.

C. Multi-step predictive accuracy across session blocks.

Line plots showing SR-based multi-step predictive accuracy as a function of predictive horizon (H = 1–5) for representative session blocks for Animal E (*top row*) and Animal F (*bottom row*). Predictive accuracy was assessed as in panel A. Performance of the SR is compared to uniform random-choice and spatially constrained random-walk reference models (black and grey lines), computed by resampling sequences separately within each session block and animal, with sequence counts matched to the empirical data. Shaded regions indicate 95% confidence intervals across cross-validation folds. Statistical comparisons against reference models were performed across predictive horizons using Monte Carlo tests with Bonferroni correction (***$p < 0.001$).

	Animal E			Animal F		
**Variable**	Coefficient	Std Error	p	Coefficient	Std Error	p
#days since last training session	0.02	0.06	1	0.06	0.09	1
experimenter	-0.06	0.37	1	-0.76	0.80	1
Session number	-0.07	0.024	0.04	-0.26	0.03	<0.001
#trials	0.03	0.11	1	-0.10	0.27	1

Table S1. Multiple linear regression assessing session-level predictors of non-rewarded events.
Related to Figure 1.
Summary of multiple linear regression models predicting the mean number of non-rewarded events per session for Animal E and Animal F. The table reports regression coefficients (β), standard errors, and Bonferroni-corrected p-values for each predictor: number of days since the previous training session, experimenter identity, session number, and number of trials performed in the session. The model accounted for 27% of the variance in non-rewarded events for Animal E ($R^2 = 0.27$) and 64% for Animal F ($R^2 = 0.64$). After Bonferroni correction for multiple comparisons, session number was the only significant predictor (Animal E: $\beta = -0.07$, $p = 0.04$; Animal F: $\beta = -0.26$, $p < 0.001$), indicating a robust decrease in non-rewarded events with experience. All other session-level covariates showed no significant contribution.

	**Animal E**		**Animal F**	
**cluster**	omissions	total visits	omissions	total visits
0	0.06 ± 0.02	8.49 ± 0.06	0.02 ± 0.01	9.65 ± 0.12
1	0.08 ± 0.04	12.97 ± 0.36	0.07 ± 0.04	17.37 ± 0.48
2	7.8 ± 0.2	0.25 ± 0.25	8.0 ± 0.0	0.0 ± 0.0
3	2.92 ± 0.26	5.58 ± 0.31	3.33 ± 0.38	7.91 ± 1.02

Table S2. Cluster centroids and clustering quality metrics.
Related to Figure 2.
Table reporting the mean (± SEM) coordinates of cluster centroids identified through unsupervised K-means clustering based on two trial-level metrics: number of omissions and total number of dispenser visits. Each trial was assigned to one of four clusters (Clusters 0–3), and centroid values are reported separately for Animal E and Animal F. To characterize the quality of the clustering solution, we report the silhouette score and inertia associated with the four-cluster partition for each animal (Animal E: silhouette = 0.73, inertia = 41.9; Animal F: silhouette = 0.68, inertia = 56.4). These values indicate good separation between clusters together with low within-cluster dispersion, supporting the identification of coherent and well-defined execution profiles. The reported centroids and clustering metrics correspond to a stable solution consistently obtained across repeated clustering runs with different initial conditions, indicating that the identified execution profiles do not depend on a particular initialization.

Predictor	Animal E β ± SE	z	p	Animal F β ± SE	z	p
Intercept	2.64 ± 0.12	22.82	<0.001	2.92 ± 0.17	17.19	<0.001
k	-0.39 ± 0.05	-8.39	<0.001	-0.81 ± 0.06	-12.91	<0.001
Session block	0.17 ± 0.07	2.47	0.013	0.78 ± 0.08	9.58	<0.001
k × block	-0.02 ± 0.03	-0.74	0.46	-0.14 ± 0.03	-4.66	<0.001
Agent (structure-only vs empirical)	-2.05 ± 0.13	-15.78	<0.001	-1.26 ± 0.19	-6.52	<0.001
k × agent	-0.24 ± 0.06	-3.92	<0.001	0.11 ± 0.08	1.44	0.151
block × agent	-0.03 ± 0.08	-0.33	0.742	-0.26 ± 0.10	-2.70	0.007
k × block × agent	0.04 ± 0.04	1.05	0.296	0.05 ± 0.04	1.38	0.166

Table S3. Logistic regression analysis of reward probability during trial progression. Related to Figure 4.

Regression coefficients (β ± SE), Wald *z* statistics, and two-tailed *p* values from logistic regression models predicting the probability of selecting a rewarded dispenser as a function of trial progression (*k*), session block, and agent identity (empirical behavior versus structure-only agent). Separate models were fitted for Animal E and Animal F. Models were fitted at the level of individual choices, with reward outcome as the dependent variable and trial progression, session block, agent identity, and their interactions as predictors. Trial progression (*k*) and session block were mean-centered prior to model fitting.

Across both animals, reward probability declined significantly with increasing *k* ($p < 0.001$), indicating robust dependence on trial progression, and was significantly lower for the structure-only agent than for empirical behavior ($p < 0.001$). Session block also had a significant positive effect in both animals (Animal E: β = 0.17 ± 0.07, p = 0.013; Animal F: β = 0.78 ± 0.08, p < 0.001), indicating higher reward probability in later session blocks.

For Animal E, the interaction between k and session block was not significant (p = 0.46), indicating that the effect of trial progression remained relatively stable across experience. However, the k × agent interaction was significant (β = -0.24 ± 0.06, p < 0.001), indicating that the dependence of reward probability on trial progression differed between empirical behavior and the structure-only agent. Neither the block × agent interaction (p = 0.742) nor the three-way interaction (k × block × agent; p = 0.296) was significant, indicating no clear evidence that the empirical advantage over the structure-only agent changed across session blocks, either overall or specifically as a function of trial progression.

For Animal F, the interaction between *k* and session block was significant (β = -0.14 ± 0.03, $p < 0.001$), indicating that the dependence of reward probability on trial progression strengthened across session blocks. In contrast to Animal E, the *k* × agent interaction was not significant ($p = 0.151$), whereas the block × agent interaction was significant (β = -0.26 ± 0.10, $p = 0.007$), indicating that the empirical advantage over the structure-only agent changed across session blocks. The three-way interaction (*k* × block × agent) was not significant ($p = 0.166$), indicating no clear evidence that the session-block-dependent modulation of trial-progression effects differed between empirical behavior and the structure-only agent.

ANALYSIS BLOCK	MEASURE(S)	STATISTICAL TEST	UNIT OF ANALYSIS	MULTIPLE-COMPARISON CORRECTION	FIGURE / TABLE
Spatial sampling of the environment	Visit counts per dispenser; omission counts per dispenser	χ^2 goodness-of-fit; post hoc two-sided binomial tests when omnibus significant	Counts pooled across trials within animal	Bonferroni (post hoc across dispensers)	Fig. 1C-D
Non-rewarded events	Mean total non-rewarded events across sessions	Linear regression (HC3 robust SE)	Session-level means	—	Fig. S1B
Non-rewarded events	Session-level covariate effects on mean non-rewarded events	Multiple linear regression (HC3 robust SE)	Session-level means	Bonferroni across predictors	Table S1
Non-rewarded events	Relative prevalence of omissions, revisits, and home returns	Kruskal-Wallis; Dunn post hoc	Session-level means and trial-level values	Bonferroni	Fig. S1C; Fig. S1E
Non-rewarded events	Covariation between non-rewarded events and sampling behavior	Pearson correlation	Session-level means and trial-level values	Bonferroni	Fig. S1D; Fig.S1F
Trial-level clustering analysis	Behavioral differences across clusters	Kruskal-Wallis; Dunn post hoc	Trial-level observations pooled within cluster	Bonferroni	Fig. S2A
Trial-level clustering analysis	Experience-dependent changes in cluster prevalence	Linear regression (HC3 robust SE)	Session-level cluster proportions	—	Fig. S2C
Transition- and sequence-based analyses	Reward rate	Mann-Whitney U (clusters); Kruskal-Wallis + Dunn (session blocks); one-sided Monte Carlo tests vs reference models	Trial-level reward-rate values; matched-N null distributions	Bonferroni	Fig. 2B; Fig. 3A
Transition- and sequence-based analyses	Global transition entropy	Permutation tests (clusters and blocks); one-sided Monte Carlo tests vs reference models	Pooled transition distributions; matched-N null distributions	Bonferroni	Fig. 2C; Fig. 3B
Transition- and sequence-based analyses	Reward rate-entropy association	Spearman rank correlation; permutation test	Session blocks	—	Fig. 3C
Transition- and sequence-based analyses	Local transition entropy heterogeneity and temporal dynamics	Permutation tests	Dispenser locations	—	Fig. S3D-E
Transition- and sequence-based analyses	Transition matrix similarity	Pearson correlation; row-wise permutation tests	Transition matrices	Bonferroni	Fig. 2D-E; Fig. S2D; Fig. S3B
Transition- and sequence-based analyses	Transition-level non-reward probability similarity	Spearman rank correlation	Shared transitions between Clusters 0 and 1	—	Fig. S2E
Transition- and sequence-based analyses	Sequence similarity within session blocks	Kruskal-Wallis; Dunn post hoc	Sequences without non-rewarded events	Bonferroni	Fig. 6F
Within-trial visit history	Reward outcome as a function of trial progression, session block, and agent identity	Logistic regression (k, block, agent, and all two-way and three-way interactions)	Choice-level observations	—	Fig. 4A-B; Fig. S4; Table S3
Markov-model analyses	Local and sequence-level predictive performance	Kruskal-Wallis + Dunn (session blocks); one-sided Monte Carlo tests vs reference models	Fold-level cross-validated predictive performance	Bonferroni	Fig. 5A, C, E, F
Successor representation analyses	Multi-step predictive performance	Kruskal-Wallis + Dunn (session blocks); one-sided Monte Carlo tests vs reference models	Fold-level cross-validated ROC AUC	Bonferroni	Fig. 7C-D; Fig. S7C

Table S4. Summary of inferential statistical analyses.
Related to STAR Methods.

All analyses were performed separately for each animal. Unless otherwise specified, statistical tests were two-sided and significance was set at $\alpha = 0.05$. Session-level analyses used 30 sessions per animal; trial-level analyses used 233 trials for Animal E and 232 trials for Animal F. Clustering analyses were performed on all trials and yielded four clusters comprising 172, 37, 12, and 12 trials for Animal E and 162, 46, 12, and 12 trials for Animal F (Clusters 0-3, respectively).

Transition- and sequence-based analyses used trials from Clusters 0 and 1 pooled by session block, resulting in a total of 209 trials for Animal E and 208 trials for Animal F (34.8 ± 2.9 and 34.7 ± 3.9 trials per block, respectively; mean ± SEM across six blocks). Sequence-based, Markov-model, and successor-representation analyses were restricted to trials without non-rewarded events: 98 eligible trials for Animal E across all six blocks and 47 for Animal F across the four blocks containing eligible trials, corresponding to 16.3 ± 2.1 and 11.8 ± 3.1 eligible trials per analyzed block, respectively (mean ± SEM).

Monte Carlo tests used one-sided empirical p-values with a +1 correction; when applicable, multiple comparisons were controlled using Bonferroni correction. HC3, heteroscedasticity-consistent robust standard errors; ROC AUC, area under the receiver operating characteristic curve.
